# Transcriptome and single-cell RNA sequencing analysis with 101 machine learning combinations and experimental verification reveals the mechanism of action of mannose metabolism in bladder cancer

**DOI:** 10.3389/fimmu.2026.1710823

**Published:** 2026-01-28

**Authors:** Anhong Li, Kaile Zhao, Tianjiao Wang, Guangyue Shi

**Affiliations:** Department of Internal Medicine-Oncology, Harbin Medical University Cancer Hospital, Harbin, China

**Keywords:** 101 machine learning, bladder cancer, mannose metabolism, prognostic genes, single-cell sequencing analysis

## Abstract

**Background:**

Bladder cancer (BLCA) is a prevalent genitourinary malignancy characterized by high recurrence and mortality rates. While mannose metabolism has demonstrated anti-tumor potential across various cancers, its role in BLCA remains underexplored. This study examines the influence of mannose metabolism on BLCA prognosis.

**Methods:**

BLCA-related datasets and genes associated with mannose metabolism (MMRGs) were obtained from public databases. Candidate genes were identified by overlapping differentially expressed genes with MMRGs. Prognostic genes were pinpointed using ten machine learning algorithms and regression analysis to develop a risk model, which was subsequently validated. A nomogram was constructed by integrating the risk score with clinical features, and its predictive accuracy was assessed. We performed functional enrichment, drug sensitivity, reverse transcription-quantitative polymerase chain reaction (RT-qPCR), Western blotting, immunohistochemistry, and immune infiltration analyses. Key cellular components were identified, and further analyses, including pathway enrichment, pseudo-temporal analysis, and cell communication, were performed.

**Results:**

CALR, SLMAP, PFKFB4, and TMTC1 were identified as prognostic genes in BLCA. Notably, the expression of SLMAP and TMTC1 was significantly downregulated in BLCA, whereas PFKFB4 and CALR were upregulated. These findings were consistently validated by RT-qPCR, Western blotting, and immunohistochemical analyses (p < 0.05). The risk model stratified patients into a high-risk group (HRG) and a low-risk group (LRG), with HRG patients exhibiting significantly poorer survival outcomes. The risk score was identified as an independent prognostic factor, and the nomogram demonstrated high diagnostic accuracy. Notable differences between HRG and LRG patients were observed in the “Ribosome” pathway. Additionally, 86 chemotherapeutic drugs exhibited significant differential responses between HRG and LRG, with 23 immune cell types showing differential abundances, including activated dendritic cells (p < 0.05). Single-cell analysis revealed macrophages as key cells in BLCA, which were classified into five subtypes, with CALR, SLMAP, and PFKFB4 influencing their expression.

**Conclusion:**

Four mannose metabolism-related prognostic genes were identified in BLCA, and macrophages were confirmed as critical cells. These findings provide valuable insights for improving prognostic assessment in BLCA.

## 1 Introduction

Bladder cancer (BLCA) is a malignant tumor originating in the bladder mucosa and ranks as one of the most common malignancies in the urinary system (1). Over 500,000 new cases and more than 200,000 deaths are reported globally each year, with a notably higher incidence in men, particularly in developed countries. Histologically, approximately 90% of BLCA cases are classified as urothelial carcinoma, with the remaining cases representing rare subtypes such as squamous cell carcinoma and adenocarcinoma. Clinically, BLCA is divided into two main categories: non-muscle invasive bladder cancer, which has a favorable prognosis but a high recurrence rate, and muscle invasive bladder cancer, which has a poor prognosis and is frequently associated with local invasion and distant metastasis (2). Current treatment options for BLCA, including surgery, chemotherapy, radiotherapy, and immunotherapy, are often inadequate in fully addressing issues of recurrence and drug resistance (3). However, existing BLCA transcriptomic classifiers exhibit significant limitations. Many models focus solely on epithelial pathways or immune contexts, neglecting the integration of mannose metabolism (metabolic flux) and O-mannosylation (protein modification), which are essential for understanding how metabolic reprogramming influences cell adhesion, matrix remodeling, and immune interactions. Furthermore, there is a lack of effective connections between bulk-level risk scores and single-cell states, such as tumor-associated macrophage–cancer-associated fibroblast (CAF) communication, which are critical for predicting therapeutic responses. Methodological transparency, including formal proportional hazards (PH) testing, calibration, and batch harmonization, is often inconsistently reported, limiting clinical confidence and reproducibility (4–7). Therefore, investigating the molecular mechanisms underlying BLCA, while developing new prognostic models and personalized treatment strategies, is essential for improving the accuracy and effectiveness of therapeutic interventions.

Mannose is a naturally occurring monosaccharide found abundantly in plants and fruits, primarily excreted *via* urine and accumulating in the bladder, where it directly impacts bladder epithelial cells and offers distinct advantages in treating urinary system diseases (8). In cancer cells deficient in key mannose-metabolizing enzymes, mannose metabolism induces energy depletion, disrupts nucleic acid metabolism, and causes genomic instability by interfering with glucose metabolism, ultimately leading to cell death (9). Enhanced mannose metabolism in BLCA regulates glycosylation processes, inhibiting PD-L1 expression and improving the response to immune therapy (10). Specifically, protein O-mannosylation mediated by endoplasmic reticulum (ER)-localized enzymes, such as TMTC1, modifies cadherins and integrins, linking glycan remodeling to cell adhesion, motility, and mechanotransduction, thereby influencing tumor invasion and interactions with the extracellular matrix (11, 12). Furthermore, high-mannose N-glycans regulate PD-L1 glycosylation, shaping tumor–host interactions and modulating the effectiveness of immune checkpoint therapies (10, 13, 14). However, further investigation is required to elucidate the precise mechanisms of mannose metabolism in BLCA and its role in disease progression.

This study used transcriptomic data from BLCA samples obtained from public databases. Differential expression analysis was first performed to identify candidate genes by comparing BLCA samples to control samples. To explore the diagnostic and prognostic significance of key genes derived from this analysis, multiple machine learning algorithms were employed for feature selection and modeling. The interpretability of these key genes was further assessed using the SHAP method, and the results were validated through reverse transcription quantitative polymerase chain reaction (RT-PCR), enabling a deeper understanding of the molecular mechanisms of these prognostic genes in BLCA. Single-cell analysis was conducted to identify critical cell populations, followed by pseudo-temporal analysis to examine the dynamic expression of prognostic genes during the differentiation of these cells. Additionally, inter-cellular communication network analysis revealed insights into tumor microenvironment (TME) interactions, providing a theoretical basis for refining treatment strategies and designing personalized therapeutic approaches for BLCA.

## 2 Materials and methods

### 2.1 Data collection

BLCA-related transcriptomic, mutation, clinical, and survival data were retrieved from the TCGA database (available at https://tcga-data.nci.nih.gov/tcga/), with the data download conducted on June 11, 2025. Specifically, the TCGA-BLCA dataset included 404 BLCA tumor-derived bladder tissue samples with corresponding survival data, along with 18 bladder tissue samples from paracancerous controls. Additionally, the GSE13507 dataset from the GEO database was incorporated into the study. Based on the GPL6102 platform, this dataset contained 165 BLCA tumor bladder tissue samples with survival data. The study also utilized the scRNA-seq dataset GSE222315 from the Gene Expression Omnibus (GEO) database (http://www.ncbi.nlm.nih.gov/geo/), built on the GPL24676 platform. Clinical information from these datasets is provided in **Supplementary Table S1**. The GSE222315 dataset included 9 BLCA tumor tissue samples and 4 paracancerous control tissue samples. A total of 130 mannose metabolism-related genes (MMRGs) were retrieved from the Molecular Signatures Database (MSigDB) (https://www.gsea-msigdb.org/gsea/msigdb) and are listed in **Supplementary Table S2**. The study workflow is depicted in **Supplementary Figure S1**.

### 2.2 Differential expression analysis

To identify differentially expressed genes (DEGs) between BLCA and control samples in the TCGA-BLCA dataset, the “DESeq2” R package (v 1.40.2) was used, with selection criteria set at an adjusted p-value < 0.05 and |log_2_ Fold Change (FC)| > 2 (15) For visualizing the results, a volcano plot was created using the “ggplot2” R package (v 3.5.2), highlighting the top 10 upregulated and downregulated DEGs, sorted in descending order based on adjusted p-values (16). The “ComplexHeatmap” R package (v 2.16.0) was employed to graphically present the expression trends of DEGs in a heatmap (17).

### 2.3 Screening and analytical evaluation of candidate genes

Candidate genes were identified by detecting overlapping genes between DEGs and MMRGs using the “ggvenn” R package (v 0.1.10) (18). These overlapping genes were considered candidate genes for further analysis. Functional annotation of the candidate genes was conducted through Kyoto Encyclopedia of Genes and Genomes (KEGG) and Gene Ontology (GO) analyses, covering biological processes (BPs), cellular components (CCs), and molecular functions (MFs), using the “clusterProfiler” R package (v 4.10.1), with a statistical cutoff of p < 0.05 (19). Furthermore, the protein–protein interaction (PPI) network for the candidate genes was constructed using the STRING database (confidence ≥ 0.15), and the network was visualized with the “circlize” R package (v 0.4.16) (20).

### 2.4 Determination of candidate key genes

To validate the list of candidate key genes, 10 machine learning algorithms were applied to both the TCGA-BLCA dataset and the GSE13507 dataset, resulting in the development of 101 predictive models. The following algorithms were used with the respective R packages: LR *via* the “glmnet” (v 4.1-8), DT *via* the “caret” (v 7.1-1), SVM *via* the “e1071” (v 1.7-16), RF *via* the “randomForest” (v 4.7-1.2), KNN *via* the “survival” (v 2.8-3), BT *via* the “xgboost” (v 1.7.10.1) (https://CRAN.R-project.org/package=xgboost), LDA *via* the “MASS” (v 7.3-60.0.1), NNET *via* the “nnet” (v 7.3-20), NB *via* the “naivebayes” (v 1.0.0), and SGBT *via* the “xgboost” (v 1.7.10.1) (21–25). A 10-fold cross-validation was performed for each method, and the optimal model was determined based on the highest AUC observed in both datasets. The genes identified through this model were considered candidate key genes.

### 2.5 Shapley Additive exPlanations model analysis

To examine the association between candidate key genes and SHAP values, SHAP analysis was performed on the TCGA-BLCA dataset using the candidate key genes and the “fastshap” R package (v 0.1.1; available *via*https://CRAN.R-project.org/package=fastshap). The results were visualized with the “Shapviz” R package (v 0.9.8) (https://github.com/ModelOriented/shapviz; https://modeloriented.github.io/shapviz/), which included a global importance plot illustrating the contribution of each candidate key gene to disease prediction, quantified by SHAP values.

### 2.6 Detection and characterization of prognostic genes

For further identification of prognostic genes, univariate Cox regression analysis (p < 0.2, Hazard Ratio [HR] ≠ 1) was conducted on candidate genes using BLCA samples with survival data from the TCGA-BLCA dataset. The “survival” R package (v 2.8-3) was employed for this analysis (26). The PH assumption test (p > 0.05) was applied to ensure the prognostic relevance of the genes. LASSO regression analysis, performed using the “glmnet” R package (v 4.1-8), was used to further refine the selection of prognostic genes (27). The model corresponding to the minimum value of lambda was chosen as the optimal model for gene selection.

### 2.7 Development and validation of a risk model

A risk model was developed based on the identified prognostic genes. The risk score was calculated using the expression levels of the prognostic genes and defined by the formula: RiskScore = ∑i Coefficient(i) * Expression of gene(i). BLCA patients in the TCGA-BLCA dataset with survival data were classified into high- and low-risk groups (HRG and LRG) based on the median risk score. Kaplan-Meier (K-M) curves and risk curves for these groups were plotted using the “survminer” R package (v 0.5.0) (28). The prognostic capacity of the risk model was evaluated using this package. To visualize the differential expression of prognostic genes, a heatmap was generated comparing HRG and LRG BLCA samples. ROC analysis was performed to assess the model’s prognostic performance for 1-, 3-, and 5-year survival, yielding AUC values ranging from 0.6 to 1. The “survivalROC” R package (v 1.0.3.1) was used for these analyses (29). The reliability of the model was further validated in the GSE13507 dataset.

### 2.8 Building and appraisal of a nomogram

To confirm the risk model as an independent prognostic factor, univariate Cox regression analysis (HR ≠ 1, p < 0.05) was conducted, incorporating risk score, age, gender, T stage, and N stage, along with the PH assumption test (p > 0.05). Subsequently, multivariate Cox regression analysis (HR ≠ 1) was performed using the “survival” R package (v 2.8-3) (30) to identify independent prognostic factors. A nomogram was constructed using the “regplot” R package (v 1.1; https://CRAN.R-project.org/package=regplot), which integrates these factors to predict 1-, 3-, and 5-year overall survival for TCGA-BLCA patients. ROC curves were plotted using the “survivalROC” R package (v 1.0.2.1), with AUC values ranging from 0.6 to 1 (29). Decision curve analysis (DCA), conducted using the “rmda” R package (v 1.6), evaluated the clinical prediction accuracy of the nomogram, with a net benefit value greater than 0 indicating a favorable prediction effect (29).

### 2.9 Immune infiltration analysis

Immune cell infiltration scores for 28 subtypes were calculated using the ssGSEA algorithm integrated in the “GSVA” R package (v 1.48.3) to assess immune cell infiltration in HRG and LRG of the TCGA-BLCA cohort (31). The Wilcoxon test was performed to determine the statistical difference in infiltration scores between HRG and LRG. Subsequent analysis focused on immune cell types with significant differences in infiltration levels (p < 0.05). Correlation analysis was conducted using the “corrplot” R package (v 0.95) (32), with thresholds of |cor| > 0.30 and p < 0.05 applied to examine associations between differentially infiltrated immune cells and prognostic genes. Stromal, immune, and ESTIMATE scores were computed for each sample using the “ESTIMATE” R package (v 1.0.13) (33), providing assessments of stromal and immune cell infiltration in the BLCA TME. Differences in scores between HRG and LRG were evaluated using the Wilcoxon test, with a p-value < 0.05 considered significant. To explore correlations among prognostic genes, risk scores, immune scores, stromal scores, and ESTIMATE scores, Spearman correlation analysis was performed using the “cor” function, with significance determined by thresholds of |cor| > 0.30 and p < 0.05.

### 2.10 Analysis of gene set enrichment

Signaling pathways related to the prognostic genes were investigated using BLCA samples from the TCGA-BLCA dataset. DEGs (HRG vs LRG) were identified using the “DESeq2” package (v 1.34.0), and ranked based on log_2_FC values. The “c2.cp.kegg.v7.4.symbols.gmt” gene set from the MSigDB database served as the reference background. Gene Set Enrichment Analysis (GSEA) was performed using the “clusterProfiler” package (v 4.10.0), assessing enrichment of the pre-ranked gene list relative to the reference set. Significance was determined by thresholds of p < 0.05 and |NES| > 1.

### 2.11 Somatic mutation analysis and drug sensitivity analysis

Mutation frequencies in HRG and LRG from the TCGA-BLCA dataset were analyzed using the “maftools” R package (v 2.18.0) (34). A waterfall plot was generated to display the top 20 genes with the highest mutation frequencies. To assess drug sensitivity in BLCA patients, IC50 values for 138 common therapeutics were obtained from the GDSC database (https://www.cancerrxgene.org/). The “oncoPredict” R package (v 1.2) was then utilized to predict these values for TCGA-BLCA samples, with significance defined as p < 0.05 (35). Drug sensitivity comparisons between HRG and LRG in TCGA-BLCA tumor samples were conducted using the Wilcoxon test, with a significance threshold of p < 0.05. Correlations between prognostic genes and the top 20 significantly different chemotherapeutic drugs were analyzed using the “corrplot” R package (v 0.95, https://github.com/taiyun/corrplot) through Spearman correlation analysis, with criteria set at p < 0.05 and |cor| > 0.3.

### 2.12 Analysis of regulatory network, GeneMANIA, chromosomal localization, subcellular localization, and expression analysis of prognostic genes

To investigate the regulatory networks of potential microRNAs (miRNAs) targeting the prognostic genes, the StarBase v3.0 database was used with a screening criterion of pancancerNum > 6. The resulting miRNA-mRNA interactions were visualized in Cytoscape. A gene-gene interaction (GGI) network was constructed using the GeneMANIA database (https://genemania.org/) to explore functional linkages among the prognostic genes. The “RCircos” R package (v 1.2.2) was utilized to visualize the chromosomal distribution of prognostic genes and map their chromosomal locations (36). Predicted scores for 5 subcellular localizations of each prognostic gene were obtained from the Deeploc database (https://services.healthtech.dtu.dk/services/DeepLoc-2.0/). Wilcoxon tests were performed to compare the expression levels of prognostic genes between BLCA tissues and normal controls from the TCGA-BLCA cohort, aiming to examine their protein expression profiles across different sample types, with significance set at p < 0.05.

### 2.13 Data quality control and cell annotation

The “Seurat” R package (v 5.30) was used to analyze the GSE222315 dataset. The following screening criteria were applied: 200 < nFeature_RNA < 6000, nCount_RNA < 20000, and percentage.mt < 10%. High variability genes (HVGs) were identified using the “Seurat” R package (v 5.30), and data normalization was performed using the same package. Principal component analysis (PCA) was conducted using the JackStraw function to derive principal component scores (37). The JackStraw function calculates the p-value for each gene in each principal component, and principal components with smaller enrichment p-values (p < 0.05) were retained. Cell classification of the GSE222315 dataset was carried out using the Seurat R package (v 5.30) with a resolution set to 0.5. The identified cell clusters were annotated using marker genes from the literature and the CellMarker database (http://xteam.xbio.top/CellMarker/) (38, 39). The marker genes selected for each cell type included: T/NK cells (NKG7, KLRD1, CD3D, CD3G), B cells (CD79A, MS4A1), macrophages (CD163, CD68, CD14), epithelial cells (EPCAM, CDH1), fibroblasts (COL1A1, COL3A1), endothelial cells (PECAM1, CD34), and mast cells (TPSAB1, KIT). A t-SNE plot was generated using the “Seurat” R package (v 5.30) to display the annotated cell clusters (37).

### 2.14 Identification of key cells

t-SNE was employed to analyze the expression patterns of prognostic genes across different cell types, aiming to explore the spatial distribution of annotated cell clusters and their expression profiles in both control and BLCA groups. Core cell types with significant differences in the expression of key prognostic genes between the patient and control groups were identified using the Wilcoxon test (p < 0.05).

### 2.15 Cell-cell communication, pseudo-temporal, and cell cycle analyses

Cell-cell communication analysis was performed using the “CellChat” R package (v 1.6.1) to examine interactions between key cell types and other annotated populations in both BLCA and control samples from the GSE222315 dataset (38). A cell-cell communication network was constructed to delineate the number and strength of interactions, as well as the degree of receptor-ligand binding. Statistical testing was conducted based on the default model. Furthermore, pseudotime trajectory analysis was conducted using the “Monocle” R package (v 2.30.1) (39). The FindAllMarkers function from the “Seurat” R package (v 5.30) was used for secondary dimensionality reduction of key cells (resolution = 0.1) (37). Dimensionality reduction during trajectory construction was performed using the reduceDimension function with the parameters set to method = ‘log’ and max_components = 2. Cell ordering was initiated automatically with the orderCells function, followed by the analysis and visualization of the cell differentiation trajectory for key cell types using the “DDRTree” R package (v 0.1.5). Differential gene analysis was conducted using the differentialGeneTest function with the model specified as fullModelFormulaStr = “~sm.ns(Pseudotime)” and parallel computation set to cores = 4. The BEAM method from the “Monocle” R package (v 2.30.1) was applied to analyze pseudotime-ordered cell data relative to a specified branch point (39). The expression dynamics of four prognostic genes along the pseudotime were visualized using the plot_pseudotime_heatmap function.

To investigate the proportion of cells in different stages of the cell cycle (G1, S, G2, and M phases), the “Seurat” R package (v 5.30) was used to examine the marker genes of the cell cycle stages in key cells and other annotated cell types (37). The CellCycleScoring function was used to score the S and G2M phases of each cell subset based on the expression of cell cycle phase marker genes. The distribution of cells across different cell cycle states was visualized using DimPlot.

### 2.16 RT-qPCR

RT-qPCR was performed to validate the expression levels of prognostic genes in clinical specimens. A total of 10 clinical tissue samples, including 5 from BLCA tumors and 5 from adjacent normal tissues, were collected at Harbin Medical University Cancer Hospital. Informed consent was obtained from all participants, and the study protocol was approved by the Ethics Committee of Harbin Medical University Cancer Hospital (Approval No. KY2024-40). Reverse transcription was carried out using the Hifair^®^ III 1st Strand cDNA Synthesis SuperMix for qPCR kit from Yeasen Biotechnology (Shanghai, China). Primer sequences are provided in **Supplementary Table S5**. qPCR assays were performed using the CFX96 Connect Real-time Quantitative Fluorescence PCR Instrument (Bio-Rad, USA). The thermal cycling conditions were as follows: initial denaturation at 95°C for 1 minute, followed by 40 cycles consisting of denaturation at 95°C for 20 seconds, annealing at 55°C for 20 seconds, and extension at 72°C for 30 seconds. Relative mRNA expression levels were calculated using the 2-ΔΔCT method. RT-qPCR data were initially exported to Excel, then imported into GraphPad Prism 10 (https://www.graphpad.com/) for statistical analysis (p < 0.05) and visualization. Primer sequences are listed in **Supplementary Table S3**.

### 2.17 Western blot analysis

Total proteins from Bladder cancer specimens and paracancerous specimens were extracted using a mixture of RIPA lysis buffer and PMSF (RIPA: PMSF = 100:1). Protein concentrations were quantified with a BCA assay kit. Equal amounts of protein were separated by 10% SDS–polyacrylamide gel electrophoresis (SDS–PAGE) and subsequently transferred onto polyvinylidene difluoride (PVDF) membranes via electrotransfer. The membranes were then washed with TBST buffer, blocked with 5% nonfat milk in TBST, and incubated overnight at 4°C with the indicated primary antibodies. After washing, membranes were incubated with horseradish peroxidase–conjugated anti-rabbit or anti-mouse secondary antibodies at room temperature. Protein bands were visualized using an enhanced chemiluminescence (ECL) detection system and imaged with a luminescent imaging workstation. Band intensities were quantified and analyzed using ImageJ and GraphPad Prism 8 software. Statistical analysis was conducted using t-tests and two-way ANOVA.

### 2.18 Immunohistochemistry

Bladder cancer tissues and adjacent normal tissues were collected from 33 patients who did not receive intravesical therapy. The cohort included 33 bladder cancer tissues and 33 matched adjacent normal tissues. Formalin-fixed paraffin-embedded (FFPE) tissue sections were departmentalized successively, re-hydrated with graded alcohols, and then thoroughly rinsed with phosphate-buffered saline (PBS) (Servicebio, China, G0002). To quench endogenous peroxidase activity, the sections were incubated in a 3% hydrogen peroxide solution (Angergech, China) at room temperature in the dark for 25 minutes. After that, the slides were immersed in PBS and washed three times on a decolorizing shaker, with each wash lasting 5min. Then, the tissues were covered with 3%BSA in the tissue chemical circle, and closed at room temperature for 30min. The slices were incubated with the specific primary antibodies targeting the prognostic genes, and then with the secondary antibodies that could bind to the detectable markers. The color developments of slices were achieved by DAB color developing solution (Servicebio, China, G1212). After staining by hematoxylin (Servicebio, China, G1040), the slices were dehydrated using a gradient alcohol concentration, and then the preserved tissues were sealed. Detailed observations were conducted under a microscope (Nikon, Japan, E100). The histochemistry scores (H-Score) were assessed by two experienced pathologists in a blind manner (14–18). H-Score (∑(pi×i) = (percentage of weak intensity cells ×1) +(percentage of moderate intensity cells ×2) +(percentage of strong intensity cells ×3).

### 2.19 Analyzing the data

Bioinformatics analysis was performed in R (v 4.2.2). Statistical comparisons in RT-qPCR and WB were conducted using the t-test, with p < 0.05 deemed statistically significant.

## 3 Results

### 3.1 Ascertainment and functions exploration of 20 candidate genes

**Figure 1:**
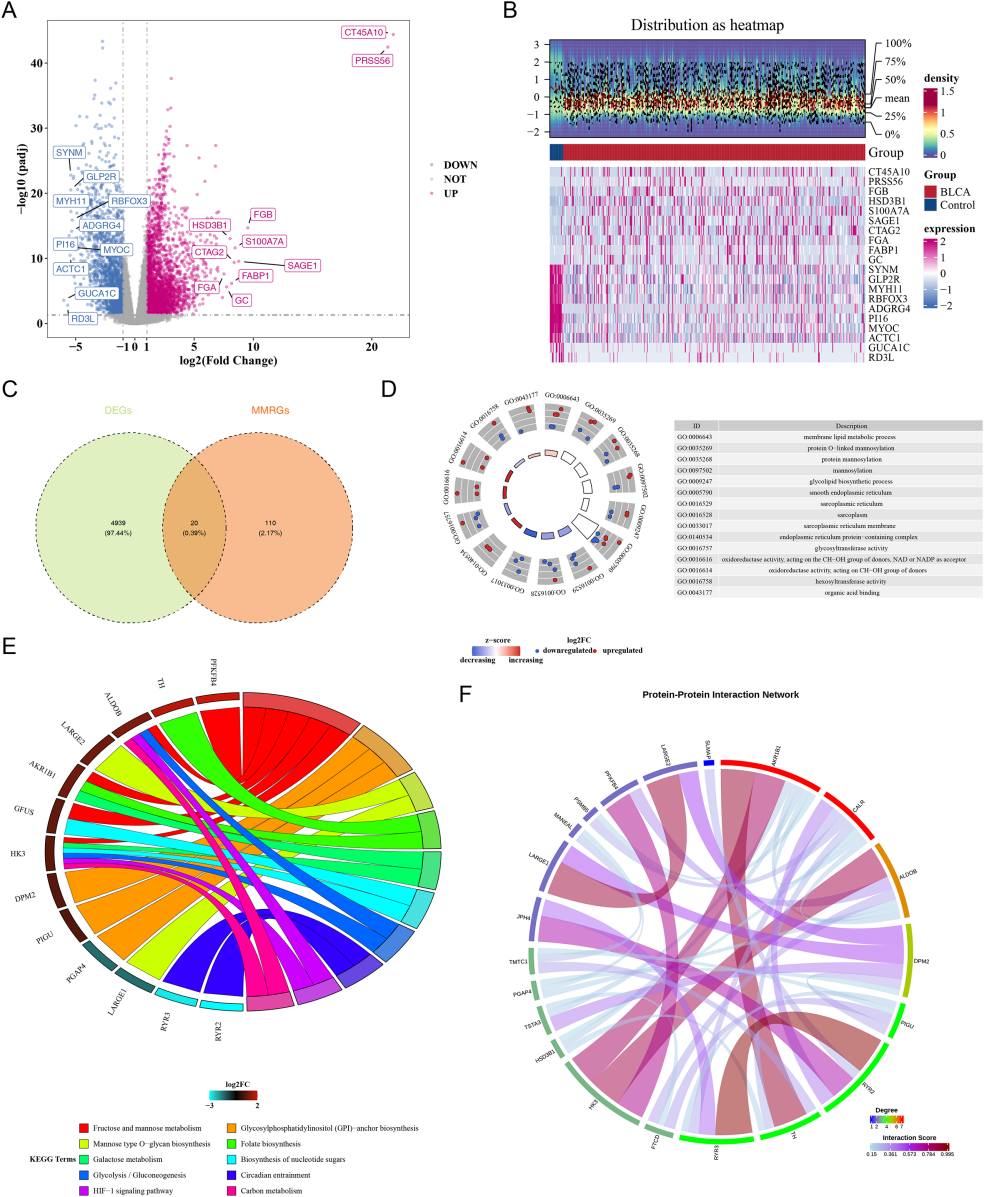
Determination of candidate genes (TCGA-BLCA dataset). **(A)** Volcanic map of DEGs. Red: up-regulated gene; Blue: down-regulated gene; Grey: Insignificant gene. **(B)** Heatmap of DEGs. Red: BLCA groups; Blue: Control groups. The color represented the expression level of the gene, and the higher the expression level, the darker the color (red indicates high expression, blue indicates low ex-pression) **(C)** Venn diagram of candidate genes. Gree: DEGs; Orange: MMRGs. **(D)** GO enrichment analysis of candidate genes. **(E)** GO enrichment analysis of candidate genes. The line represented correlation. **(F)** PPI network. The lines represented their interactions, and the blue nodes represented genes.

A comprehensive analysis of the TCGA-BLCA dataset identified 4,959 DEGs, including 2,940 up-regulated and 2,019 down-regulated genes in BLCA samples (**Figures 1A, B**). The intersection of these 4,959 DEGs and 130 MMRGs revealed 20 candidate genes (**Figure 1C**). Functional annotation identified 434 significant GO terms (p < 0.05), encompassing biological processes (e.g., “mannosylation”; 344 terms), cellular components (e.g., “sarcoplasm”; 26 terms), and molecular functions (e.g., “glycosyltransferase activity”; 64 terms) (**Figure 1D**; **Supplementary Table S4**). Additionally, 14 KEGG pathways, such as “Carbon metabolism,” were significantly enriched (p < 0.05) (**Figure 1E**; **Supplementary Table S5**). The Protein-Protein Interaction (PPI) network analysis revealed interactions among the remaining 20 genes (**Figure 1F**).

### 3.2 Identification and analysis of 11 candidate key genes

**Figure 2:**
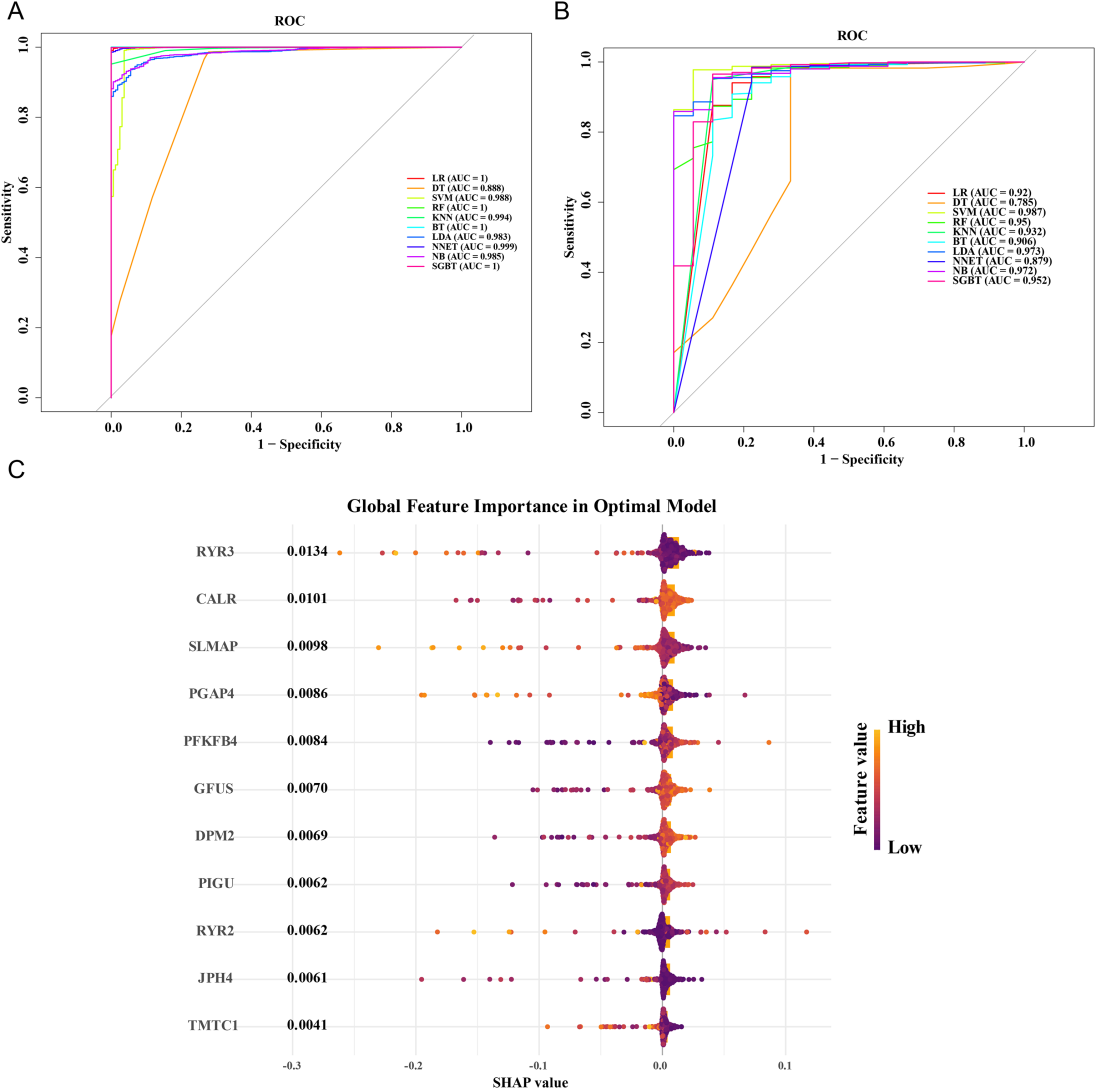
Identification and analysis of candidate key genes. **(A)** ROC curves of 10 ma-chine learning algorithms in TCGA-BLCA dataset. **(B)** ROC curves of 10 machine learning algorithms in GSE13507 dataset. The closer the AUC was to 1, the stronger the predictive ability. **(C)** HAP algorithm identifies candidate genes (TCGA-BLCA dataset). The vertical axis represented each candidate gene in the SVM model, the horizontal axis position and point color represented the SHAP value of the corresponding gene in the sample, and the bar graph represented the im-portance score of the gene.

In the TCGA-BLCA dataset, evaluation of ten algorithmic models based on Receiver Operating Characteristic (ROC) curve analysis demonstrated that the support vector machine (SVM) algorithm achieved the highest area under the curve (AUC) score. As a result, the SVM model was selected as the optimal algorithm, with AUC values of 0.988 in the TCGA-BLCA dataset (**Figure 2A**) and 0.987 in the GSE13507 dataset (**Figure 2B**). The following genes identified by the SVM model were defined as candidate key genes: PFKFB4, CALR, RYR3, SLMAP, GFUS, DPM2, PIGU, PGAP4, JPH4, RYR2, and TMTC1. SHAP analysis indicated that RYR3 had the greatest impact on model predictions, with a higher gene content significantly increasing the prediction result. Genes with a SHAP value > 0 were deemed relatively important, resulting in the final inclusion of 11 candidate key genes in the analysis (**Figure 2C**).

### 3.3 Development and validation of a high-performance risk model based on 4 prognostic genes

**Figure 3:**
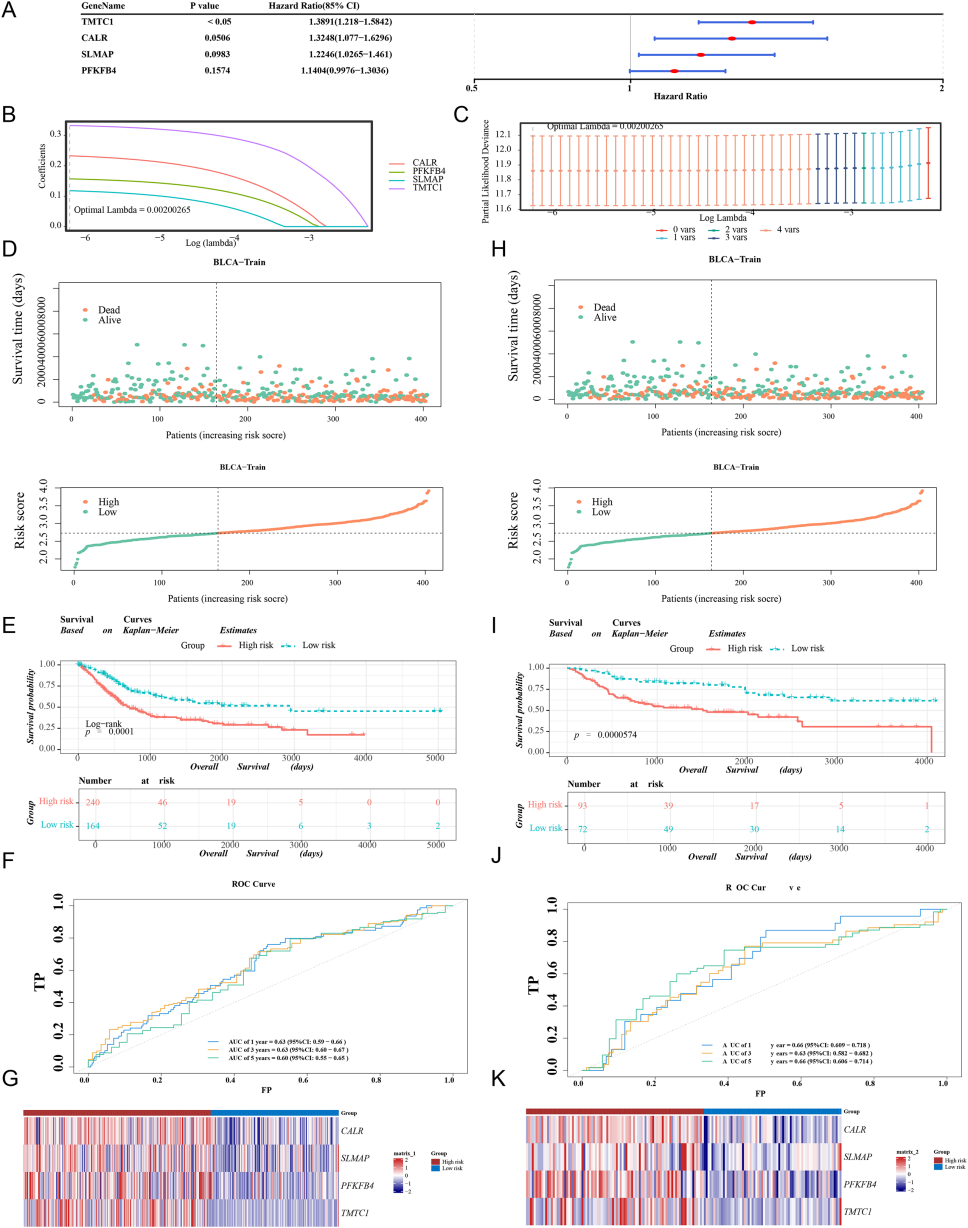
Development and Validation of Risk Model. **(A)** Univariate Cox regression analysis to identify genes associated with prognosis. The red dot represented the HR value, and the line segmented on both sides indicate the 95% confidence interval of the HR value (HR represented the hazard ratio, HR value>1 was a risk factor, and HR value<1 was a protective factor) (TCGA-BLCA dataset). **(B)** LASSO logic coefficient penalty diagram. Different colors represented different genes (TCGA-BLCA dataset). **(C)** Cross validation error curve graph. Each line represented a gene. As lambda increases, the variable coefficient of genes tends towards 0. When reaching the optimal lambda, remove variables with coefficients equal to 0. Optimal lambda selection with minimum lambda value (TCGA-BLCA dataset). **(D)** Survival Status Distribution Map and Risk Curve in TCGA-BLCA dataset. Orange: Dead; Green: Alive. Orange: High groups; Green: Low group. **(E)** Kaplan Meier survival curve plot in TCGA-BLCA dataset. Red: High risk groups; Blue: Low risk groups. **(F)** ROC curve in training set in TCGA-BLCA dataset. The closer the AUC was to 1, the stronger the predictive ability. **(G)** Heatmap of prognostic genes in high and low-risk groups in TCGA-BLCA dataset. Red: High risk groups; Blue: low risk groups. The color represented the expression level of the gene, and the higher the expression level, the darker the color (red indicates high expression, blue indicates low expression) **(H)** Survival Status Distribution Map and Risk Curve in GSE13507 dataset. Orange: Dead; Green: Alive. Orange: High groups; Green: Low group. **(I)** Kaplan Meier survival curve plot in GSE13507 dataset. Red: High risk groups; Blue: Low risk groups. **(J)** ROC curve in training set in GSE13507 dataset. The closer the AUC was to 1, the stronger the predictive ability. **(K)** Heatmap of prognostic genes in high and low-risk groups in GSE13507 dataset. Red: High risk groups; Blue: low risk groups. The color represented the expression level of the gene, and the higher the expression level, the darker the color (red indicates high expression, blue indicates low expression).

In the TCGA-BLCA dataset, candidate prognostic genes were identified through univariate Cox regression, with four genes meeting the criteria (HR ≠ 1, p < 0.05; **Figure 3A**) and satisfying the PH assumption (p > 0.05; **Supplementary Figure S2**). Four prognostic genes—CALR, SLMAP, PFKFB4, and TMTC1—were selected using the least absolute shrinkage and selection operator (LASSO) method, with a minimum lambda of 0.00200265 as the threshold (**Figures 3B, C**). A risk prediction model was constructed using these four genes. Using a median risk score of 2.724771 as the cutoff, the model stratified 404 BLCA patients from the TCGA-BLCA dataset into HRG (n = 240) and LRG (n = 164) groups. Similarly, a median risk score of 8.297005 was used to stratify 165 BLCA patients from the GSE13507 dataset into HRG (n = 93) and LRG (n = 72). Risk curves and survival status plots in the TCGA-BLCA cohort clearly differentiated the two risk groups, with HRG demonstrating increased mortality and shorter survival time (**Figure 3D**). K-M analysis revealed significantly lower survival probabilities for HRG patients in the TCGA-BLCA cohort (p = 0.0001; **Figure 3E**). ROC curve analysis showed favorable predictive accuracy in the TCGA-BLCA dataset, with AUC values exceeding 0.6; the AUC values for 1-, 3-, and 5-year survival were 0.63, 0.63, and 0.60, respectively (**Figure 3F**). CALR, SLMAP, PFKFB4, and TMTC1 exhibited higher expression levels in HRG (**Figure 3G**). These findings were further validated in the GSE13507 dataset (**Figures 3H–K**), supporting the effectiveness of the risk model in stratifying BLCA patients.

### 3.4 Construction of a nomogram from prognostic factors

**Figure 4:**
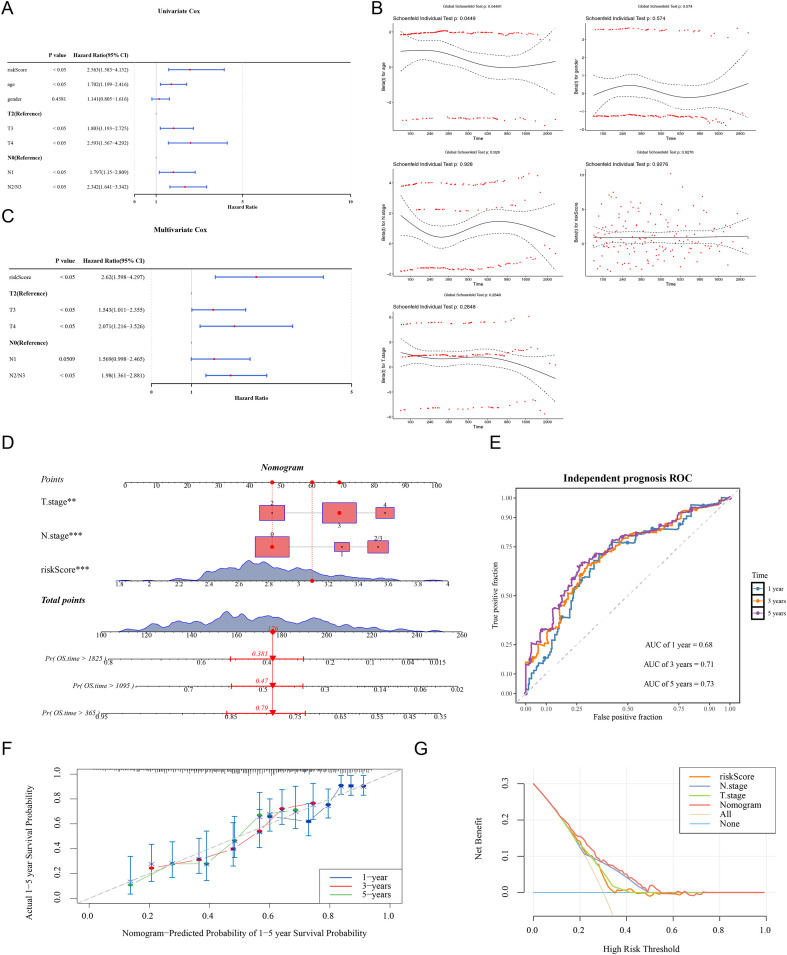
Construction of Nomogram (TCGA-BLCA dataset). **(A)** Univariate Cox regression analysis to identify genes associated with prognosis. The red dot represented the HR value, and the line seg-ments on both sides indicate the 95% confidence interval of the HR value (HR represents the hazard ratio, HR value>1 was a risk factor, and HR value<1 was a protective factor). **(B)** Single factor pH test. Red dots indicated different time points, while blue dashed lines indicated confidence intervals (CIs). **(C)** Multivariate Cox analysis of forest plots. Hazard Ratio refers to the risk allocation ratio. A Hazard Ratio greater than 1 carries a higher risk, while a Hazard Ratio less than 1 carries a lower risk. **(D)** Nomogram model. Score: Single item score represents the single item score corresponding to each variable at different values; The total score represents the sum of the individual scores corresponding to all variable values. Pre-diction probability: represents the probability of survival. **(E)** ROC curve of nomogram. The closer the AUC was to 1, the stronger the predictive ability. **(F)** Calibration curve of nomogram. Different colors represented different years. **(G)** DCA curve of nomogram. The line segment represented the net return at each risk threshold, the blue line parallel to the horizontal axis (None line) represented that all samples are negative and no intervention is ap-plied, so the net return of intervention is 0. The diagonal line (All line) represented that all samples were positive and all individuals receive the net return after intervention. Momo-gram represented the overall net benefit of the prediction model within the entire range (0–1).

Univariable Cox regression (**Figure 4A**) and PH assumption testing (**Figure 4B**; p > 0.05) in the TCGA-BLCA dataset confirmed that risk score, N stage, and T stage were suitable for further multivariable analysis (**Figure 4C**), establishing them as significant prognostic factors for BLCA. A nomogram was developed to predict disease progression (**Figure 4D**). The model, based on a total risk score of 176 derived primarily from the key prognostic factor, risk score, demonstrated robust predictive performance, with AUC values of 0.68, 0.71, and 0.73 for 1-, 3-, and 5-year predictions, respectively (**Figure 4E**). Calibration curves showed a close alignment with reference curves, confirming the accuracy of the nomogram for predictions (**Figure 4F**). Additionally, DCA indicated a high net benefit of the model (**Figure 4G**). In conclusion, the developed nomogram exhibited strong prognostic performance for predicting survival in BLCA patients, supporting its potential application in personalized risk assessment and clinical decision-making.

### 3.5 Immune microenvironment in BLCA patients

**Figure 5:**
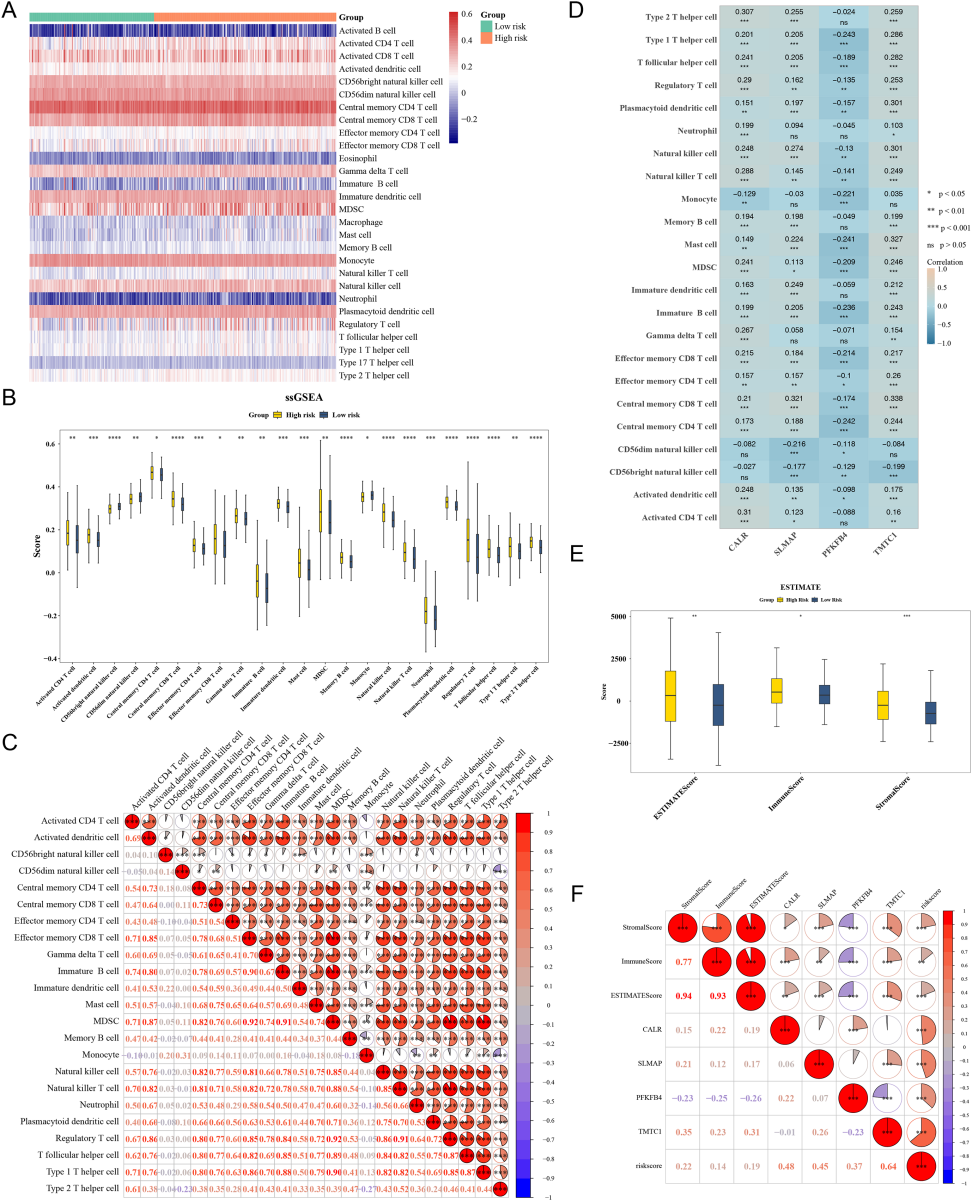
Immune microenvironment (TCGA-BLCA dataset). **(A)** Immune cell heatmap in high-risk and low-risk groups. Green: Low risk group; Red: High risk group. The color represented the expression level of the gene, and the higher the expression level, the darker the color (red indicates high expression, blue indicates low expression). **(B)** Differences in immune cells between high-risk and low-risk groups. Yellow: High risk groups; Blue: Low risk groups. *P<0.05; **P<0.01; ***P<0.001; ****P<0.0001. **(C)** Correlation heatmap between differential immune cells. Red represented positive correlation, the stronger the correlation, the redder the color, and blue represented negative correlation, the stronger the correlation, the bluer the color. *P<0.05; **P<0.01; ***P<0.001; ****P<0.0001. **(D)** Correlation heatmap between key genes and differential immune cells. *P<0.05; **P<0.01; ***P<0.001; ****P<0.0001. **(E)** Differences in Immune Score, Matrix Score, and ESTIMATE Score between High and Low Risk Groups. Yellow: High risk groups; Blue: Low risk groups. *P<0.05; **P<0.01; ***P<0.001; ****P<0.0001. **(F)** Correlation heatmap between prognostic genes and immune score, ma-trix score, and ESTIMATE score. Red represented positive correlation, the stronger the corre-lation, the redder the color, and blue represents negative correlation, the stronger the cor-relation, the bluer the color. *P<0.05; **P<0.01; ***P<0.001; ****P<0.0001. **(D)** Cor-relation heatmap between key genes and differential immune cells. *P<0.05; **P<0.01; ***P<0.001; ****P<0.0001.

Immunity plays a pivotal role in the initiation and progression of BLCA[15]. In the TCGA-BLCA dataset, immune infiltration analysis revealed that activated CD4 T cells accounted for the highest proportion in HRG patients (**Figure 5A**). Additionally, 23 immune cells showed differential abundance between HRG and LRG, including activated dendritic cells, which were more abundant in HRG patients (**Figure 5B**; p < 0.05). Correlation analysis showed the highest correlation between effector memory CD8 T cells and MDSCs (cor = 0.92, p < 0.001) (**Figure 5C**). TMTC1 exhibited the strongest positive correlation with central memory CD8 T cells (cor = 0.338, p < 0.05) (**Figure 5D**). Among HRG patients, stromal, immune, and ESTIMATE scores were all higher (**Figure 5E**), indicating a stronger immune response in HRG samples. A statistically significant correlation was observed between TMTC1 and StromalScore (r = 0.35, p < 0.05). All four prognostic genes showed significant positive correlations with the risk score, with TMTC1 demonstrating the strongest association (r = 0.64, p < 0.05) (**Figure 5F**).

### 3.6 Tumor mutation and drug sensitivity in HRG and LRG of BLCA patients

**Figure 6:**
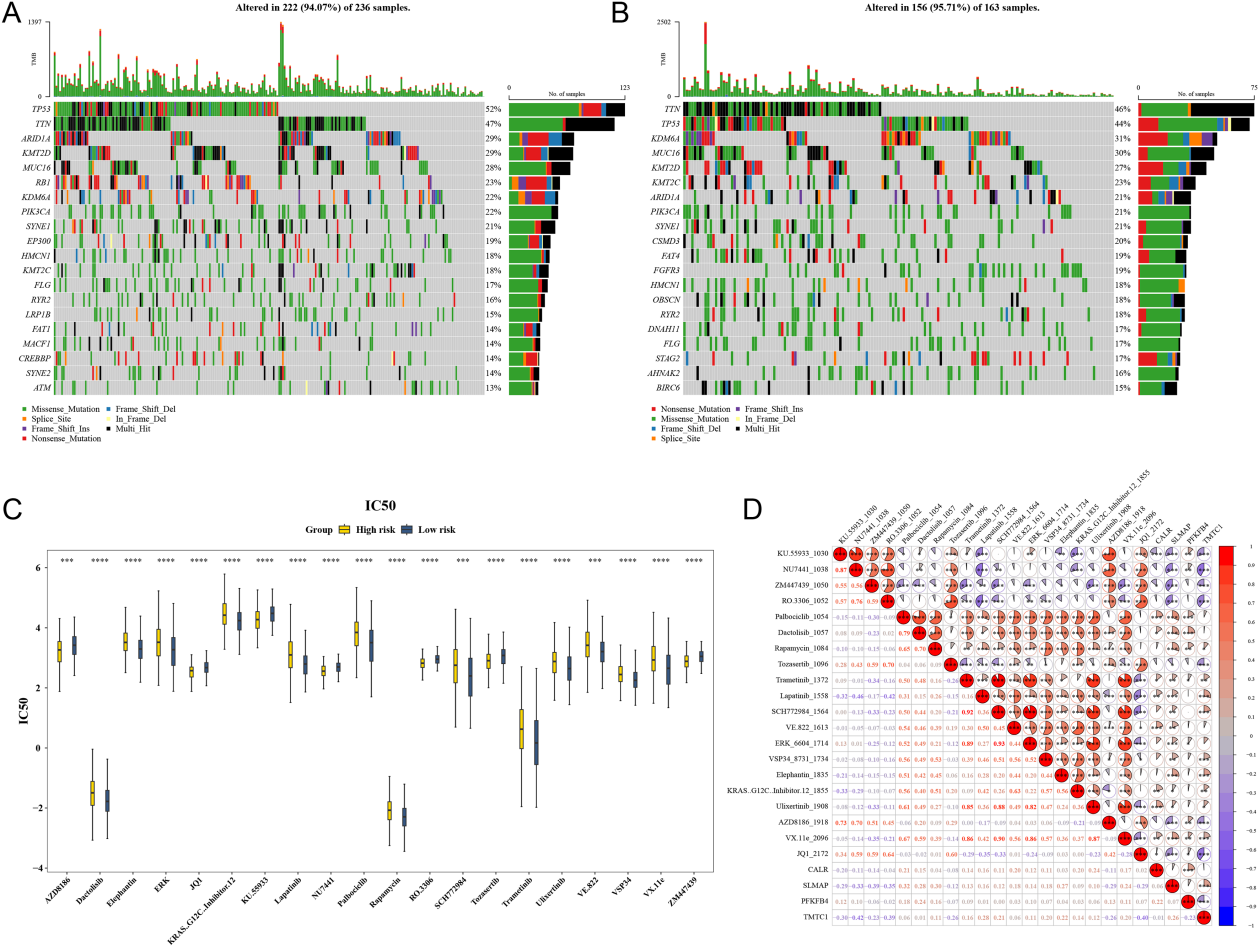
Tumor mutation and drug sensitivity (TCGA-BLCA dataset). **(A)** Analysis of Somatic Cell Mutations in High Risk Group Tumors. **(B)** Analysis of Somatic Cell Mutations in Low Risk Group Tumors. Different colors represented different invariant types. **(C)** Differences in IC50 values of drugs between high-risk and low-risk groups. Yellow: High risk groups; Blue: Low risk groups. *P<0.05; **P<0.01; ***P<0.001; ****P<0.0001. **(D)** Correlation heatmap between chemotherapy drugs, prognostic genes, and chemotherapy drugs. Red represented positive cor-relation, the stronger the correlation, the redder the color, and blue represented negative correlation, the stronger the correlation, the bluer the color. *P<0.05; **P<0.01; ***P<0.001; ****P<0.0001.

Mutation analysis in the TCGA-BLCA dataset identified the top five most frequently mutated genes in both HRG and LRG. Genes such as TTN and TP53 were significantly mutated in both groups (**Figures 6A, B**). In HRG, missense mutations were the most common, while multi_Hi mutations predominated in LRG. Chemotherapeutic drug sensitivity analysis revealed significant differences in sensitivity between HRG and LRG patients for 86 drugs (p < 0.05) (**Figure 6C**). The top 20 drugs, such as Dactolisib, Elephantin, and ERK, exhibited highly significant disparities in sensitivity (p < 0.001). A significant negative correlation was found between the TMTC1 gene and NU7441 (cor = -0.42, p < 0.05), as well as between Palbociclib and SLMAP (cor = -0.32, p < 0.05) (**Figure 6D**), suggesting potential therapeutic effects of these drugs on BLCA.

### 3.7 Comprehensive functional analysis of 4 prognostic genes

**Figure 7:**
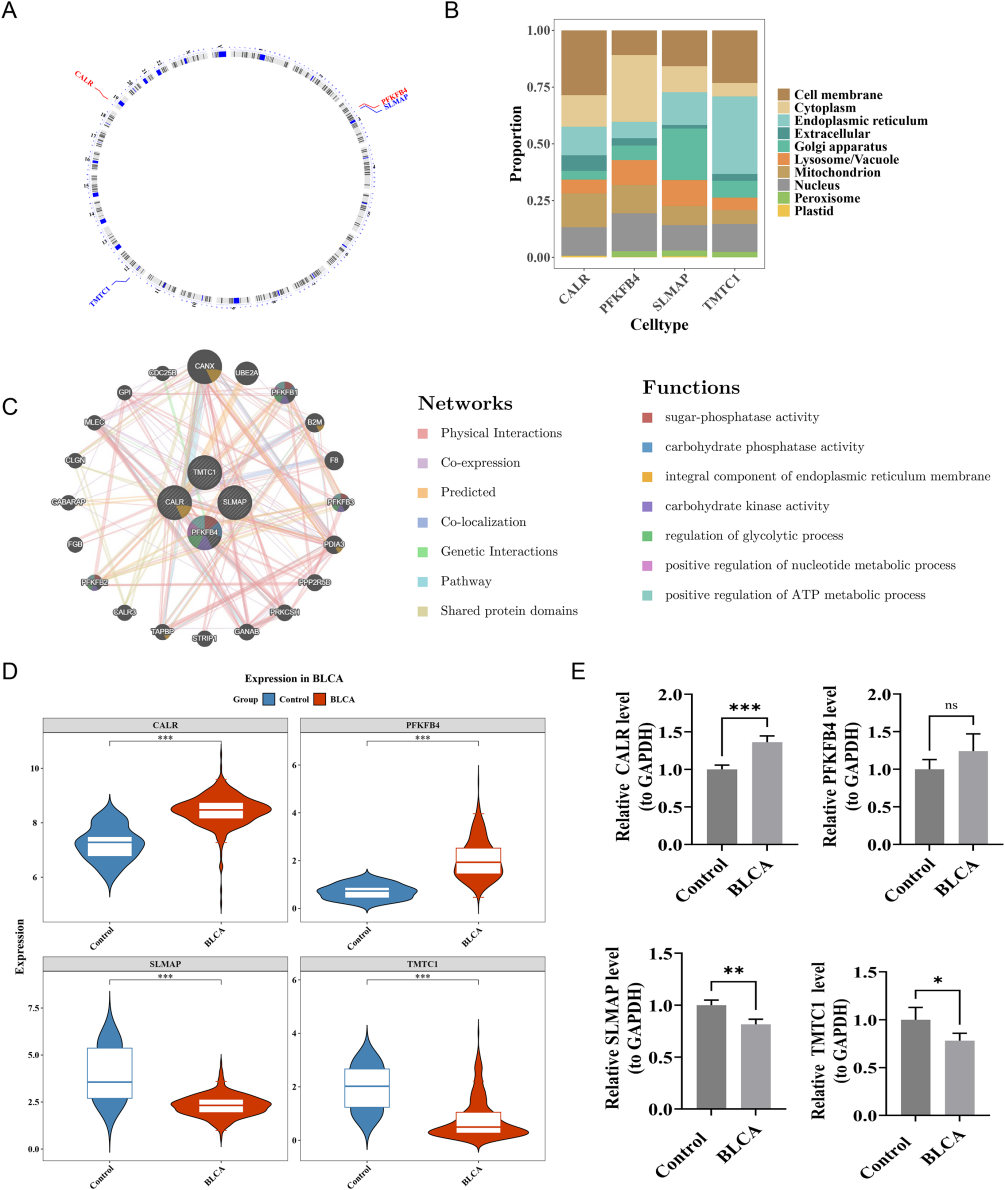
Comprehensive functional analysis of prognostic genes. **(A)** Chromosome Localization Analysis of Prognostic Genes (TCGA-BLCA dataset). **(B)** Subcellular localization of prognostic genes. Different colors indicated different subcellular localization (TCGA-BLCA dataset). **(C)** GGI network. The central circle represented 4 prognostic genes, while the outer circle represents 20 other genes related to prognostic genes. The thickness of the lines indicated the strength of their interactions, with thicker lines indicating stronger interactions. The color of the lines represented different interactions (TCGA-BLCA dataset). **(D)** Prognostic gene expression level analysis (TCGA-BLCA dataset). Blue: Control groups; Red: BLCA groups. ***P<0.001. **(E)** RT-qPCR validation of the four prognostic genes (CALR, SLMAP, PFKFB4, and TMTC1) in BLCA tumor tissues and adjacent normal tissues. (*p < 0.05; **p < 0.01; ***p < 0.001; ns, not significant).

**Figure 8:**
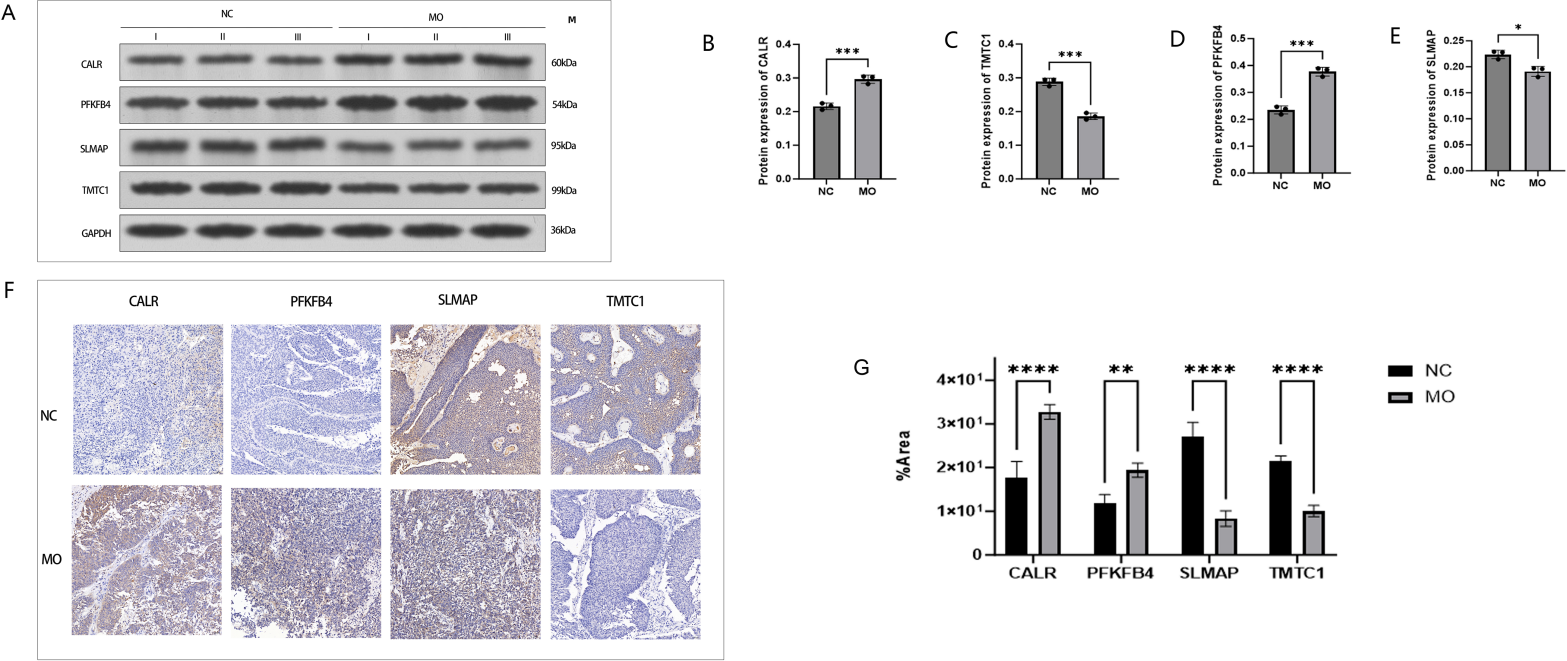
Validation of mannose metabolism–related protein expression in bladder cancer tissues. **(A)** Western blot analysis showing expression levels of CALR, PFKFB4, SLMAP, and TMTC1 in tumor tissues (MO) and adjacent normal tissues (NC), with GAPDH as a loading control. **(B–E)** Western blot expression of CALR, PFKFB4, TMTC1, and SLMAP in normal and tumor tissues. Among them, MO is the tumor tissue group, and NC is the adjacent normal tissue group. **(F)** Immunohistochemical (IHC) staining for IHC staining quantification of prognostic genes (CALR, PFKFB4, SLMAP, and TMTC1) in tumor and paracancerous tissues. **(G)** Box plots of H‐score quantification for IHC staining quantification of prognostic genes (ANLN, DSG1, FBN2, MAP1A, and SVIL) in tumor and paracancerous tissues. One asterisk (*) indicates p < 0.05; three asterisks (***) indicate p < 0.0001; a greater number of asterisks denotes a higher level of statistical significance.

In the TCGA-BLCA dataset, chromosomal localization analysis indicated the positions of PFKFB4 and SLMAP on chromosome 3, TMTC1 on chromosome 12, and CALR on chromosome 19 (**Figure 7A**). Further analysis revealed that CALR had the highest expression in the cell membrane, PFKFB4 in the cytoplasm, SLMAP in the Golgi apparatus, and TMTC1 in the ER (**Figure 7B**). GeneMANIA database analysis revealed that the four prognostic genes interacted with 20 other genes, exhibiting distinct biological functions. For instance, PFKFB4 was involved in sugar-phosphatase activity (**Figure 7C**). In the TCGA-BLCA dataset, PFKFB4 and CALR showed significantly higher expression levels in tumor tissues, while TMTC1 and SLMAP exhibited significantly lower expression compared to normal tissues (p < 0.05) (**Figure 7D**). Similarly, RT-qPCR analysis showed that in tumor tissues, the expression of CALR was significantly upregulated (p < 0.05), while PFKFB4 expression also exhibited an upward trend (although not statistically significant, p > 0.05), and the expression of SLMAP and TMTC1 was significantly downregulated (p < 0.05) (**Figure 7E**). Western blot analysis revealed that the protein levels of CALR and PFKFB4 were significantly upregulated in tumor tissues compared with adjacent normal tissues (P < 0.05), whereas TMTC1 and SLMAP were notably downregulated (P < 0.05) (**Figures 8A–E**). IHC staining demonstrated that CALR and PFKFB4 was significantly upregulated in the tumor group(P < 0.05), while SLMAP and TMTC1 was prominently downregulated in the tumor group(p<0.05) (**Figures 8F, G**). These findings were consistent with the previous analytical results.

### 3.8 Exploration of pathways and regulatory networks in BLCA

**Figure 9:**
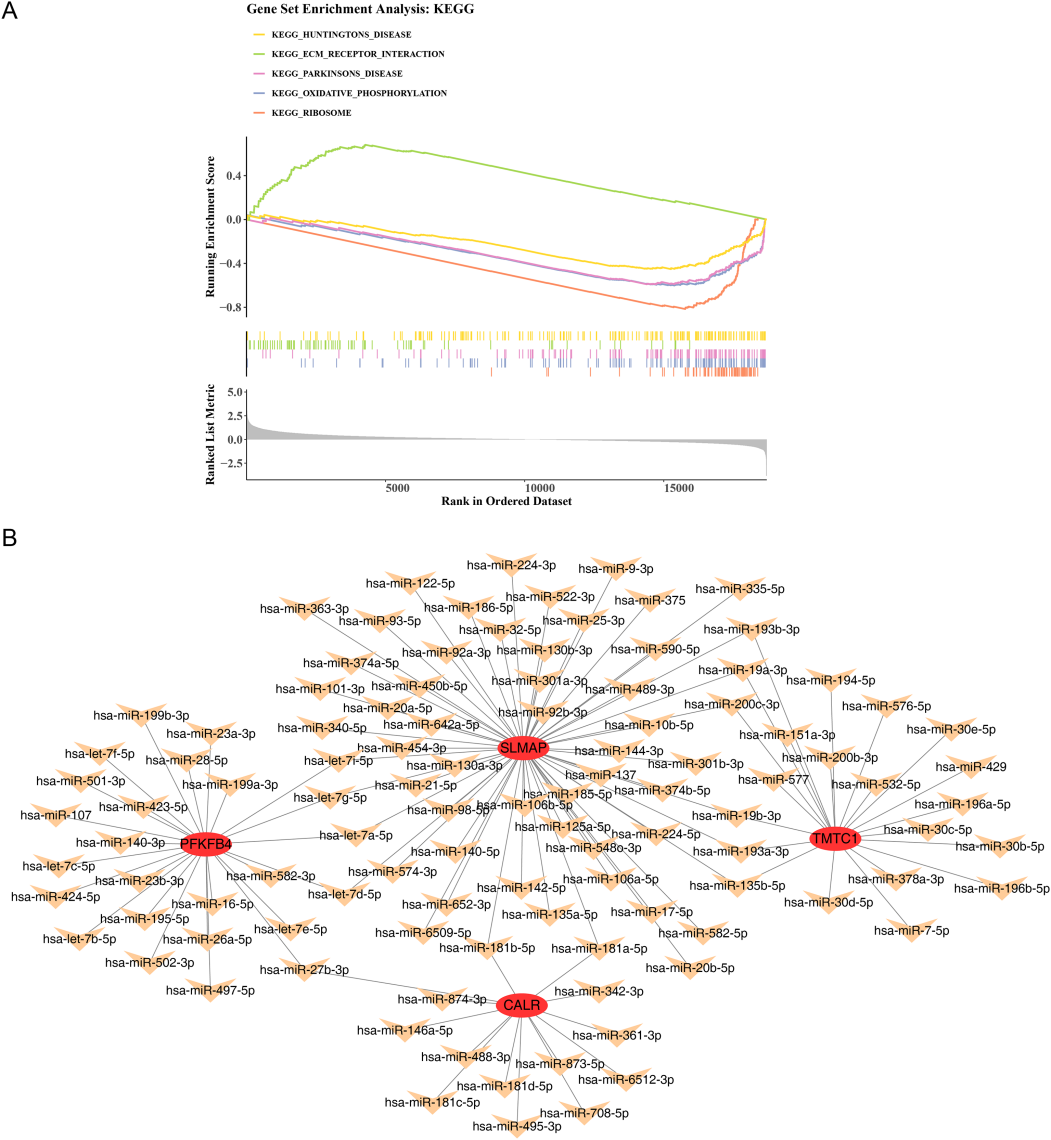
Exploration of pathways and regulatory networks in BLCA (TCGA-BLCA dataset). **(A)** GSEA enrichment analysis. Part 1: The top five lines were the line chart of gene Enrichment Score. The peak of the line on the upper side indicates significant enrichment in the upregulated path-way, while the peak of the line on the lower side indicates significant enrichment in the downregulated pathway. The vertical axis represented the corresponding Running ES, and there was a peak in the line graph, which was the Enrichment score of this gene set. The genes before the peak are the core genes under this gene set. The horizontal axis represented each gene in this gene set, corresponding to the vertical line resembling a barcode in the second part. Part 2: The barcode like part, called Hits, where each vertical line corresponded to a gene in the gene set. Part 3: The rank value distribution map for all genes, with the vertical axis being the ranked list metric, which is the value of the gene’s ranking quantity. It can be understood as the “FC value after formulaic processing”. **(B)** lncRNA-miRNA-mRNA network. The red circle represented prognostic genes, the orange ar-row represented miRNA, and the green arrow represented lncRNA.

In the TCGA-BLCA dataset, a total of 65 pathways were identified between HRG and LRG. Among these, five pathways were selected for visualization, including the top one significantly upregulated pathway and the top four significantly downregulated pathways. The upregulated pathway was ECM receptor interaction, while the significantly downregulated pathways included oxidative phosphorylation (**Figure 9A**, **Supplementary Table S6**) (p < 0.05, |NES| > 1). These results suggest that these pathways contribute to the pathogenesis of BLCA.

In the miRNA-mRNA network, for example, hsa-miR-27b-3p-PFKFB4, CALR interacted with 14 miRNAs, SLMAP with 59 miRNAs, PFKFB4 with 25 miRNAs, and TMTC1 with 21 miRNAs (**Figure 9B**). These results provide insight into the potential regulatory roles of these biomarkers during the evolution of BLCA.

### 3.9 Exploring cell enrichment pathways and identification of macrophages as key cells in BLCA

**Figure 10:**
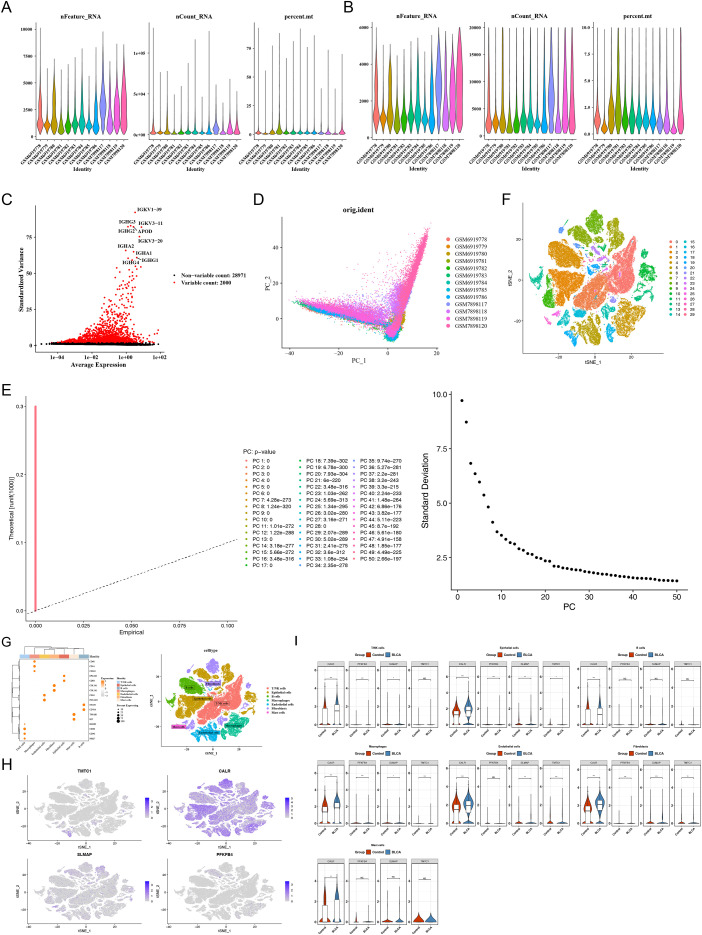
Identification of key cells (GSE222315dataset). **(A, B)** Single cell data quality control before and after. NFeature-RNA represents the number of gene expressions in cells, and excessive or insufficient cell expression indicates poor cell quality and needs to be removed; NCount_SNA rep-resents the count of a gene. If the count was too high, it indicates that the gene is overex-pressed, which can introduce significant errors and needs to be removed; Percent_tt indicates the proportion of mitochondria, and the size of the mitochondrial proportion reflects the degree of cell damage. The higher the degree of cell damage, the larger the mitochondrial proportion. **(C)** Screening of highly variable genes. The red dots represented the 2000 highly variable genes selected, while the black dots represent other genes. **(D)** PCA sample cell distribution map. Points represent cells. **(E)** Principal component analysis Jackstrand plot and inflection point plot. **(F)** Cell tSNE clustering diagram. Different colors represented different clusters. **(G)** DotPlot plot after cell annotation. The darker the color, the higher the expression. **(H)** Cell tSNE clustering diagram. Different colors represented different clusters. **(I)** Distribution map of key gene expression in different cell types.

The GSE222315 dataset contained data from 102,232 cells and 30,971 genes (**Figure 10A**). After data quality control, the total number of cells was adjusted to 91,301, while the number of genes remained 30,971 (**Figure 10B**). Subsequently, 2,000 highly variable genes (HVGs) were identified, with the top 10 HVGs highlighted (**Figure 10C**). PCA showed a relatively uniform distribution between the samples and groups, with no detectable batch effects (**Figure 10D**). The standard deviation of the first 30 principal components (PCs) demonstrated significant changes, with the trend weakening beyond the 30th PC. Thus, the top 30 PCs were selected (p < 0.05) (**Figure 10E**). t-SNE analysis revealed that the cells clustered into 30 distinct clusters (**Figure 10F**). These clusters were subsequently annotated into seven cell types (**Figure 10G**). To identify cell types that differentially express the four prognostic genes, we performed Wilcoxon tests comparing BLCA samples versus controls across all annotated cell types. The results showed that macrophages, along with endothelial cells and fibroblasts, exhibited significant differences in the expression of these prognostic genes (p < 0.05). In addition, given the established central role of tumor-associated macrophages in BLCA progression and tumor microenvironment remodeling as reported in prior studies (16, 17), we focused subsequent analyses on macrophages as a key cellular compartment.

### 3.10 Exploring the interactions, differentiation, and cell cycle of macrophages

**Figure 11:**
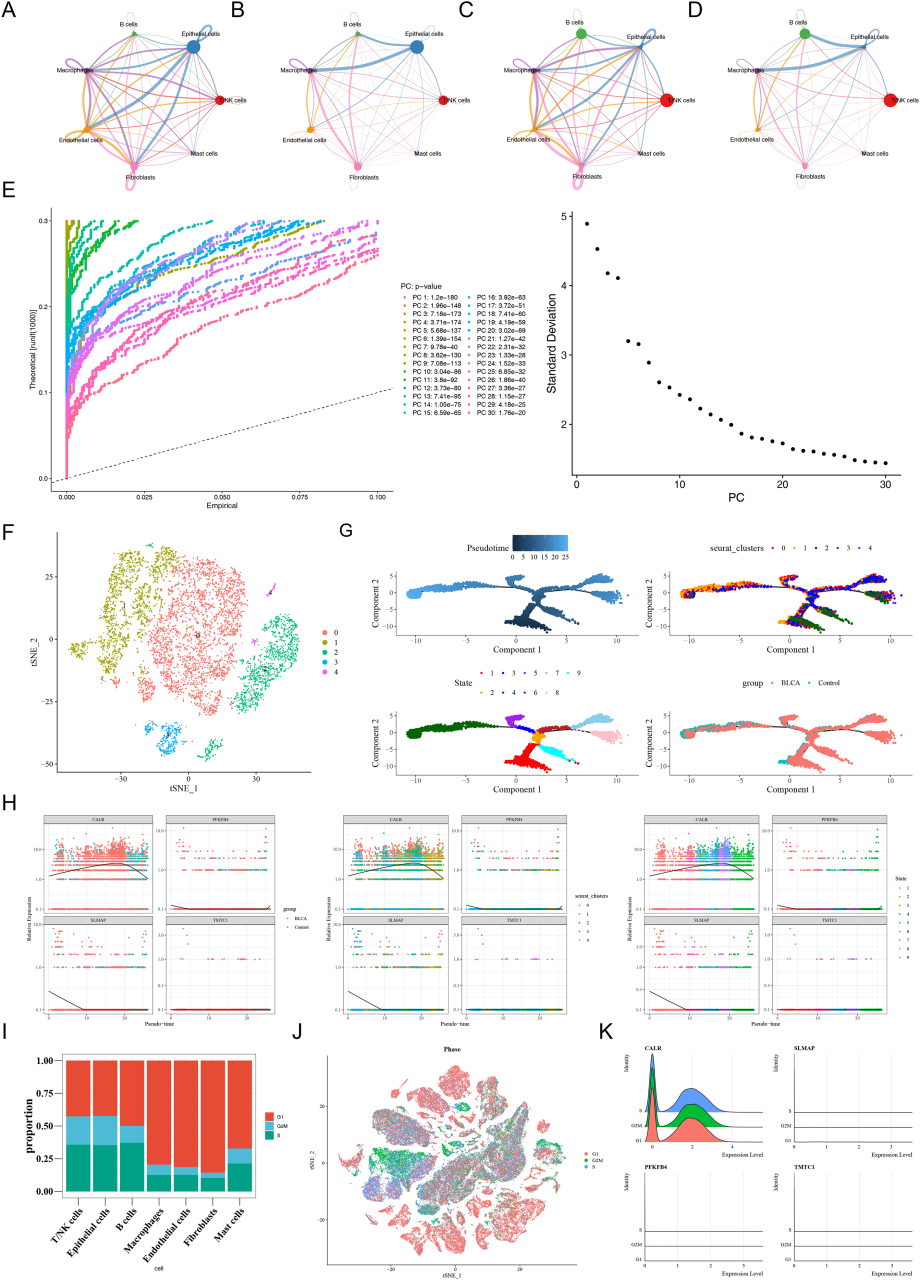
Exploring the interactions, differentiation, and cell cycle of macro-phages (GSE222315dataset). **(A)** Number of intercellular connections Control group. **(B)** Number of intercellular connections BLCA group. **(C)** Inter cell weight network diagram Control group. **(D)** Inter cell weight network diagram BLCA group. The size of the dot rep-resented the number of cells. The thickness of intercellular connections represented the total interaction strength or density between cells. **(E)** PCA analysis results of single-cell dataset. **(F)** Cluster analysis results. Each point represented a cell. **(G)** Time trajectory diagram for time series analysis. **(H)** Distribution of gene expression of key genes in macrophages during pseudo time. **(I)** Proportion of key cell cycles. **(J)** Cell cycle of different cell types. **(K)** The expression of prognostic genes at different stages of the cell cycle.

In the GSE222315 dataset, cell-to-cell communication analysis revealed frequent interactions between macrophages and fibroblasts in BLCA (**Figures 11A, B**). However, the frequency and number of interactions between these cell types decreased (**Figures 11C, D**), suggesting that the enhancement of their interaction may be associated with the pathological progression of BLCA. Further analysis of macrophages using secondary dimensionality reduction selected the first 20 PCs (**Figure 11E**), revealing five distinct subclusters (**Figure 11F**). Pseudo-temporal analysis of macrophages identified nine differentiation stages, showing a progressive trajectory from an early (dark blue) to a more mature (light blue) state. Subcluster 3 represented the earliest stages of differentiation, while subcluster 1 marked the final stage (**Figure 11G**). The prognostic gene TMTC1 remained relatively stable throughout development, while SLMAP and PFKFB4 exhibited high expression in the early stages before stabilizing. CALR showed consistently high expression, initially increasing and then decreasing (**Figure 11H**). These results suggest that CALR, SLMAP, and PFKFB4 are primarily involved in the expression of key cells. Cell cycle analysis indicated that the G1 phase predominated in macrophages (**Figure 11I**), with a strong overlap among the three phases, suggesting minimal impact from the cell cycle on the single-cell data (**Figure 11J**). The prognostic genes SLMAP, PFKFB4, and TMTC1 showed no significant variation across different phases. In contrast, CALR exhibited lower expression in the G1 phase and higher levels in the S and G2M phases, implying its potential role in regulating cell cycle progression (**Figure 11K**).

## 4 Discussion

The molecular mechanisms of mannose metabolism in tumors, such as BLCA, have garnered significant research interest. Previous studies have shown that mannose metabolism can suppress PD-L1 expression and enhance immunotherapeutic responses by regulating glycosylation processes (10). However, the specific mechanisms through which this metabolic pathway influences tumor prognosis in BLCA remain incompletely understood. This study further investigates this issue. From a technical standpoint, rather than relying on generalized algorithmic strategies, this research employed optimized machine learning approaches—such as SVM combined with SHAP interpretation—to enhance both the accuracy and interpretability of the prognostic model. Additionally, single-cell RNA sequencing technology allows for precise profiling of gene expression at the single-cell level, revealing cellular heterogeneity. This is essential not only for understanding the complex pathogenic mechanisms of BLCA but also for providing critical methodological support in identifying key cell populations and describing the dynamic expression patterns of prognostic genes (40, 41).

In this study, mannose-pathway genes were prioritized by integrating differential expression analysis (TCGA-BLCA dataset), feature importance (SVM model with SHAP interpretation, TCGA-BLCA/GSE13507 datasets), and survival association (univariate Cox regression, TCGA-BLCA dataset). These analyses were further distilled into a compact signature that remained a reliable prognostic factor alongside T and N stage. The four genes reflect complementary axes of tumor fitness. CALR, an ER chaperone and damage-associated molecular pattern, links proteostasis stress to tumor antigenicity and phagocytic clearance, aligning with immune-active TME states and better response potential (42). PFKFB4 regulates glycolytic/PPP flux to preserve NADPH and counteract ROS under stress, supporting metabolically aggressive BLCA and correlating with poorer prognosis. TMTC1, an ER O-mannosyltransferase for cadherins/integrins, enhances adhesion/FAK signaling and matrix engagement, promoting invasion and stromal remodeling (12). SLMAP, a membrane-anchored scaffold that interfaces with Ca²^+^ handling and STRIPAK–Hippo control, likely coordinates proliferation–migration coupling and disease progression (43). These axes explain why the CALR–PFKFB4–TMTC1–SLMAP signature correlates with macrophage-rich TME programs and effectively stratifies BLCA survival.

Functional readouts, including GSEA and immune infiltration analyses, align with these roles. High-risk tumors exhibit enrichment in ribosome/translation, oxidative phosphorylation, ECM–receptor interactions, and PI3K–AKT–mTOR signaling, reflecting metabolic plasticity and matrix remodeling that facilitate invasion and immune evasion (44–48). Immune deconvolution and ESTIMATE scores indicate a denser, more suppressive stromal-immune environment in the high-risk group, which is characterized by macrophage- and fibroblast-biased ecosystems and altered T-cell states.Using single-cell transcriptomic analysis, we systematically evaluated the cellular localization of four prognostic genes within the tumor microenvironment (TME). Wilcoxon rank-sum tests comparing bladder cancer (BLCA) and control samples identified macrophages as one of the most significantly differentially expressed cell types. Given the established role of tumor-associated macrophages (TAMs) in BLCA progression (16, 17), we selected this lineage for detailed downstream analysis.Notably, in specific macrophage subpopulations, the expression patterns of genes such as TMTC1 and SLMAP diverged from bulk tissue observations or predicted risk directions. This discrepancy underscores the pronounced heterogeneity of macrophages (49–51) and the dynamic nature of gene regulation (52). Pseudotime analysis supported this, revealing that CALR, SLMAP, and PFKFB4 expression varied according to macrophage differentiation stages. Specifically, SLMAP and PFKFB4 levels peaked during early differentiation, while CALR expression correlated with cell cycle progression.These findings indicate that the biological roles of these genes are state-dependent. Consequently, single-cell resolution does not undermine their prognostic value; rather, it provides a nuanced understanding of their functions through the lens of cell-state regulation. Future integration with spatial transcriptomics may further clarify these subpopulation-specific roles.

Orthogonal RT-qPCR in clinical specimens validated these trends at the tissue level: tumors showed higher CALR expression (P < 0.05) compared to controls, a directionally consistent but non-significant increase in PFKFB4 expression, and significantly lower expression of SLMAP and TMTC1 (both P < 0.05). These patterns align with the model’s biological framework—CALR upregulation corresponds with ER-stress/UPR engagement, while the reduced expression of TMTC1 and SLMAP suggests attenuation of O-mannosylation and adhesion–trafficking programs that facilitate ECM remodeling and macrophage–CAF crosstalk (12, 43, 47, 53). Notably, disease-versus-control differences and within-tumor prognostic gradients capture distinct axes; thus, the downregulation of TMTC1 and SLMAP in tumors does not preclude their positive association with risk within tumors, and the modest increase in PFKFB4 likely reflects cohort size, stromal admixture, and state heterogeneity captured by single-cell trajectories. Consistent with these pathway nodes, drug-response inference reveals differential sensitivity patterns (e.g., correlations between DNA-PK inhibition readouts and TMTC1 levels), suggesting rational combinations with DNA damage response (DDR)–targeting agents or metabolic modulators (54, 55). These findings support a model where mannose-connected proteostasis and glycolysis shape a macrophage-interfacing TME that influences clinical outcomes.

These experimental findings align with and expand upon three active research areas. First, previous studies have shown that limiting mannose catabolism (e.g., reduced phosphomannose isomerase [PMI] activity) makes tumor cells more vulnerable, with exogenous mannose or PMI depletion potentially remodeling energy metabolism and enhancing radiosensitivity (9, 56, 57). By linking mannose-related gene programs to clinical prognosis (*via* our four-gene signature), TME composition (*via* single-cell and ESTIMATE analyses), and pathway wiring (*via* GSEA), our data translate these mechanistic insights into BLCA-specific prognostic biomarkers, namely the CALR/SLMAP/PFKFB4/TMTC1 signature. In addition to mannose catabolism, our findings align with and expand on prior research into glycosylation, proteostasis stress, and antitumor immunity. Second, work on glycosylation, proteostasis stress, and antitumor immunity has established CALR as a key immunogenic-cell-death signal and ER chaperone that integrates the unfolded-protein response (UPR), antigenicity, and phagocytosis. Bladder-focused UPR signatures have also been shown to stratify outcomes and therapeutic responses. Our signature’s CALR/ER-centric axis and UPR-adjacent enrichments are consistent with these findings, while TMTC1 links mannose utilization directly to O-mannosylation of adhesion molecules, with pan-cancer evidence supporting prognostic and immune associations (58–60). High-mannose N-glycans can also influence tumor-host interactions and modulate checkpoint efficacy by impacting PD-L1 glycosylation (10, 61). Third, our results are consistent with and extend recent single-cell research on TME hubs in BLCA. Single-cell atlases and communication analyses increasingly highlight the role of macrophage and CAF hubs in muscle-invasive bladder cancer progression, with fibroblast signatures correlating with T-cell infiltration and immune contexture. Our macrophage-centric communication patterns and stromal enrichments in the high-risk group mirror these findings, positioning mannose-linked genes within similar ecological niches (61, 62). Notably, PFKFB4—another core gene in our signature—further links to this TME ecosystem by mediating cancer metabolic rewiring and hypoxia resilience. Its regulation of glycolysis likely provides the metabolic foundation for the macrophage-biased TME observed in high-risk groups (63). Additionally, PI3K–AKT–mTOR pathway enrichment from GSEA aligns with well-established urothelial oncogenic circuitry and ongoing therapeutic exploration (4, 64).

This study focuses on mannose metabolism in BLCA and establishes a comprehensive research framework by examining four prognostic genes (CALR, SLMAP, PFKFB4, and TMTC1). It connects CALR-mediated ER proteostasis, TMTC1-regulated O-mannosylation, and PFKFB4-involved glycolytic regulation with the macrophage-enriched TME and BLCA prognosis, addressing the mechanistic gap linking these processes. This evidence-based academic framework also provides a foundation for subsequent methodological analyses and translational studies. Methodologically, this systems-based approach is facilitated by robust design: the SVM–SHAP pipeline, combined with single-cell anchoring, offers a generalizable strategy for distilling metabolism-wide priors into minimal, interpretable predictors.

Building on this academic and methodological foundation, the translational value is clear. The nomogram and risk groups could refine selection for perioperative therapy or surveillance intensity, as well as inform prospective trials testing DDR inhibitors, PI3K/mTOR agents, or metabolic interventions (e.g., mannose supplementation in PMI-stratified contexts). The connection to macrophage–CAF circuits supports rational combinations that co-target metabolic fitness and immunosuppression, such as pairing DDR or PI3K pathway inhibitors with macrophage reprogramming strategies.

**Figure 12:**
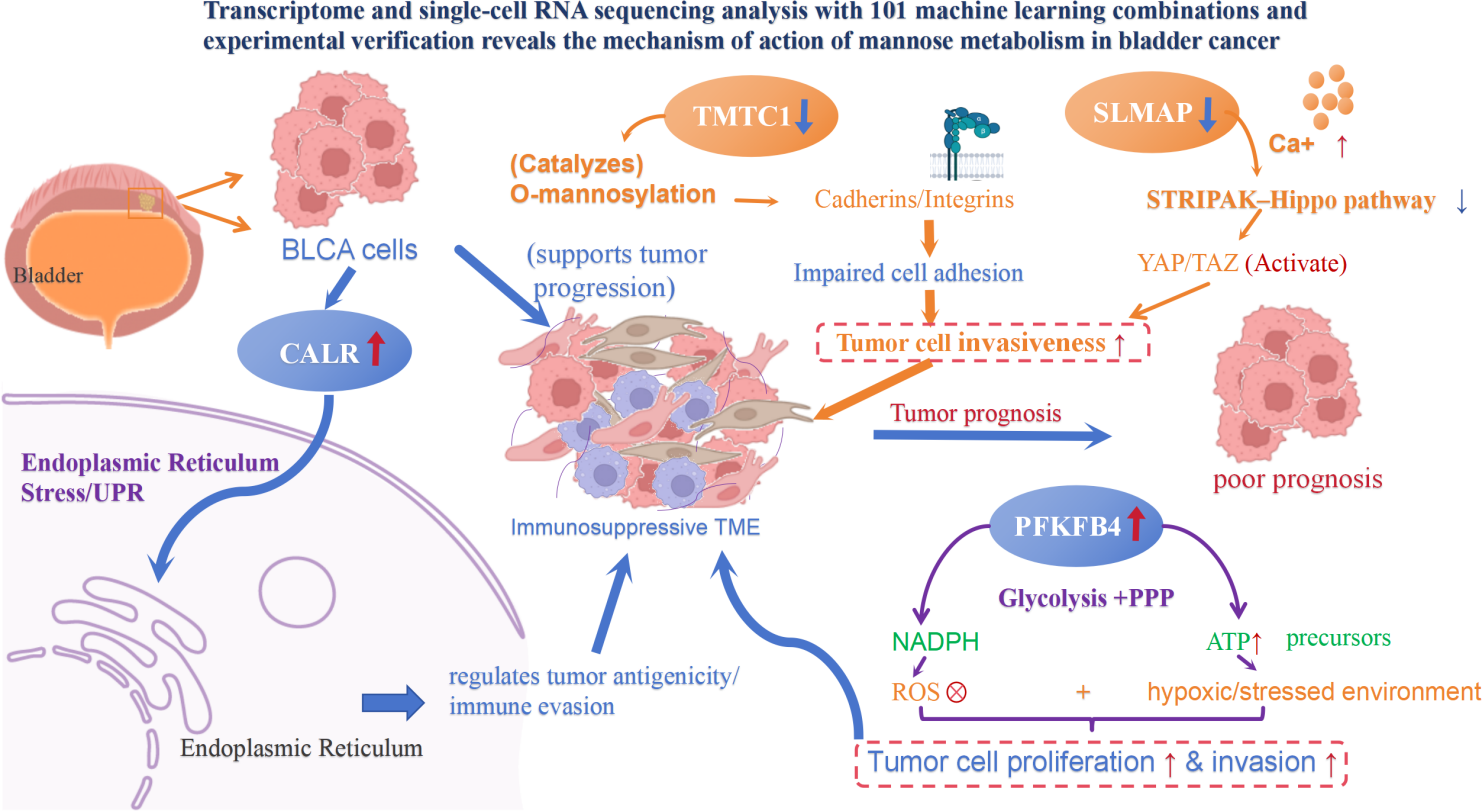
Schematic diagram illustrating the synergistic mechanism of the four mannose metabolism-related genes in BLCA. CALR regulated tumor antigenicity and the immune microenvironment via ER stress/UPR. TMTC1 catalyzed O-mannosylation of cadherins/integrins, weakening cell adhesion and promoting tumor invasion. SLMAP modulated calcium signaling and the STRIPAK–Hippo pathway, activating YAP/TAZ to enhance proliferation–migration coupling. PFKFB4 maintained NADPH levels and redox homeostasis through the glycolysis/PPP pathway, providing energy for tumor proliferation/invasion. Together, these four genes synergistically shaped an immunosuppressive TME, ultimately promoting malignant progression and poor prognosis in BLCA.

In the complex pathogenesis of BLCA, the four genes CALR, SLMAP, PFKFB4, and TMTC1 likely do not function in isolation but rather collaborate to drive tumor progression through a synergistic and complementary mechanism. Functionally, PFKFB4 regulates glycolysis and the pentose phosphate pathway to maintain NADPH levels, which helps tumor cells preserve REDOX homeostasis under metabolic stress and provides the energy required for rapid proliferation (65). As an ER chaperone, CALR not only participates in the UPR to maintain protein homeostasis, but also enhances tumor antigen presentation and translocation in response to immunogenic cell death (ICD), thus regulating the immune response within the TME (66, 67). TMTC1 modulates tumor cell interactions with the extracellular matrix by modifying adhesion molecules like cadherins and integrins, promoting local invasion and matrix remodeling (12). SLMAP, as a membrane-anchored scaffold protein, is involved in calcium signal transduction and regulation of the STRIPAK-Hippo pathway, coordinating cell proliferation and migration (68). These genes may form functional complementarities across critical biological axes—energy metabolism (PFKFB4), protein homeostasis and immunity (CALR), cell adhesion and invasion (TMTC1), and proliferation and migration synergy (SLMAP)—together constructing a multi-dimensional network that supports the malignant evolution of BLCA. In summary, CALR, SLMAP, PFKFB4 and TMTC1 form a synergistic network by regulating multiple biological axes including metabolism, protein modification, cell adhesion and signal transduction, thereby collectively promoting the malignant progression of BLCA (**Figure 12**). Future research should explore the interactions between these four genes in BLCA through experimental validation to enhance the understanding of the disease’s pathogenesis.

In summary, this study identified a four-gene signature (CALR, SLMAP, PFKFB4, TMTC1) centered on mannose metabolism, which mechanistically links three fundamental biological processes—protein homeostasis (mediated by CALR), glycolysis (mediated by PFKFB4), and adhesion-related glycosylation (mediated by TMTC1)—and is associated with a macrophage-enriched TME status. This signature effectively distinguishes survival outcomes in BLCA patients. The robustness of this signature is supported by multiple lines of evidence: single-cell analysis confirmed its consistency with TME cellular interaction patterns, RT-qPCR validated the gene expression trends in clinical specimens, and the nomogram constructed from this signature enabled individualized risk assessment. At the protein level, Western blotting and immunohistochemistry analyses further confirmed the gene expression patterns observed in clinical specimens;which were consistent with the bioinformatics results. These findings reaffirm the reliability of the biomarker in predicting bladder cancer prognosis. Collectively, the multidimensional validation indicates that the four-gene mannose metabolism–related signature is biologically robust and clinically relevant, offering a promising tool for prognostic assessment in bladder cancer patients.

This study has several limitations. First, the retrospective design of public datasets may introduce potential biases, which could affect the robustness of prognostic models. Additionally, the small clinical sample size used for RT-qPCR verification limits statistical power. This is particularly true for genes with weak effects, such as PFKFB4, where achieving statistical significance is challenging, thus somewhat limiting the generalizability of the findings. Second, while this study focused primarily on the correlation between genes and prognosis, the causal roles of CALR, SLMAP, PFKFB4, and TMTC1 in cell proliferation, invasion, metabolic reprogramming, or immune regulation in BLCA models have not been validated through functional gain or loss experiments. Furthermore, drug sensitivity predictions were based on computational models and have not yet been experimentally verified in BLCA-specific organoids or patient-derived xenograft (PDX) models. To address these limitations, future research will aim to expand the sample size and conduct multi-center, large-scale prospective cohort studies to validate the clinical efficacy of the identified biomarkers and the robustness of the prognostic model. Advanced technologies such as gene editing, metabolic flux analysis, and spatial transcriptomics will be employed to further explore the functional mechanisms of these key genes in cell and animal models. Additionally, interventional research based on PMI/mannose metabolism will be pursued, examining its potential in combination therapy with DDR inhibitors, PI3K/mTOR pathway targets, and others. Ultimately, the goal is to transition from mechanistic exploration to clinical precision diagnosis and treatment, thereby offering more effective strategies for BLCA management.

## 5 Conclusions

This study presents a mechanistically interpretable four-gene signature centered on mannose metabolism (PFKFB4, CALR, TMTC1, and SLMAP), which effectively stratifies survival outcomes in patients with BLCA. The signature, constructed and validated using support vector machine–Shapley additive explanation (SVM–SHAP) analysis in the TCGA-BLCA and GSE13507 cohorts, is supported by clear mechanistic connections: PFKFB4 regulates glycolysis, CALR mediates ER proteostasis, TMTC1 modulates O-mannosylation of adhesion molecules, and SLMAP coordinates calcium signaling with proliferation–migration processes. Integration with GSE222315 single-cell data further revealed that this signature is associated with macrophage-enriched TMEs, with macrophages identified as key cells in BLCA (p < 0.001), as well as stroma-related programs. Multivariate Cox regression analysis confirmed that the signature provides independent prognostic value beyond T stage and N stage (HR = 1.32, p < 0.001). RT-qPCR analyses of clinical specimens from Harbin Medical University Cancer Hospital validated these findings: RT-qPCR analysis showed that in tumor tissues, the expression of CALR was significantly upregulated, while PFKFB4 expression also exhibited an upward trend (although not statistically significant), and the expression of SLMAP and TMTC1 was significantly downregulated, further substantiating the translational potential of this signature from transcriptomic analysis to biomarker application. By linking metabolic flux (*via* PFKFB4), O-mannosylation (*via* TMTC1), and immune–stromal interactions (*via* CALR/SLMAP), this study establishes a clinically applicable tool and proposes verifiable translational directions, including prospective validation across muscle-invasive and non-muscle-invasive BLCA subtypes, as well as early clinical trials of metabolism–immunity combination therapies (such as PI3K/mTOR inhibitors combined with macrophage reprogramming strategies or DDR inhibitors related to TMTC1, such as NU7441, in combination regimens).

## Data Availability

The datasets presented in this study can be found in online repositories. The names of the repository/repositories and accession number(s) can be found in the article/**Supplementary Material**.

## Supplementary material

Supplementary Figure 1 Analysis flowchart.

Supplementary Figure 2 PH test chart (TCGA-BLCA dataset).

Supplementary Table 1 List of clinical information.

Supplementary Table 2 Summary table of MMRGs.

Supplementary Table 3 Summary table of primers.

Supplementary Table 4 Summary table of GO analysis of candidate genes (TCGA-BLCA dataset).

Supplementary Table 5 Summary table of KEGG analysis of candidate genes (TCGA-BLCA dataset).

Supplementary Table 6 Summary table of GSEA analysis (TCGA-BLCA dataset).
