# Supplementary figures and images for: Transcriptome and single-cell RNA sequencing analysis with 101 machine learning combinations and experimental verification reveals the mechanism of action of mannose metabolism in bladder cancer
Source: Front Immunol. 2026 Jan 28;17:1710823. doi: 10.3389/fimmu.2026.1710823 (PMC12891155; doi:10.3389/fimmu.2026.1710823)

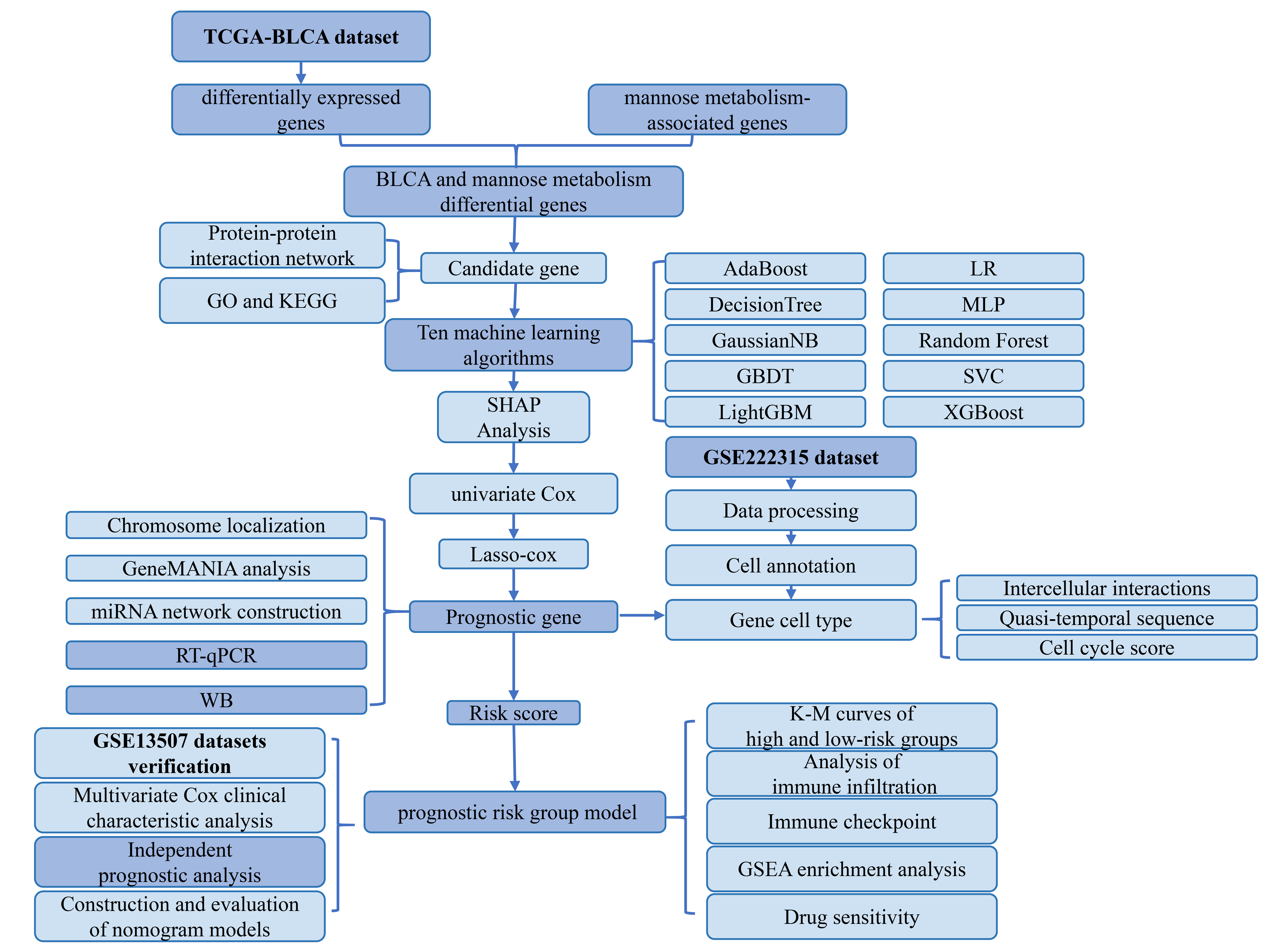

Supplement: Supplementary Figure 1 — Analysis flowchart. [file DataSheet1.zip › Supplementary Information/Figure S1.tif]

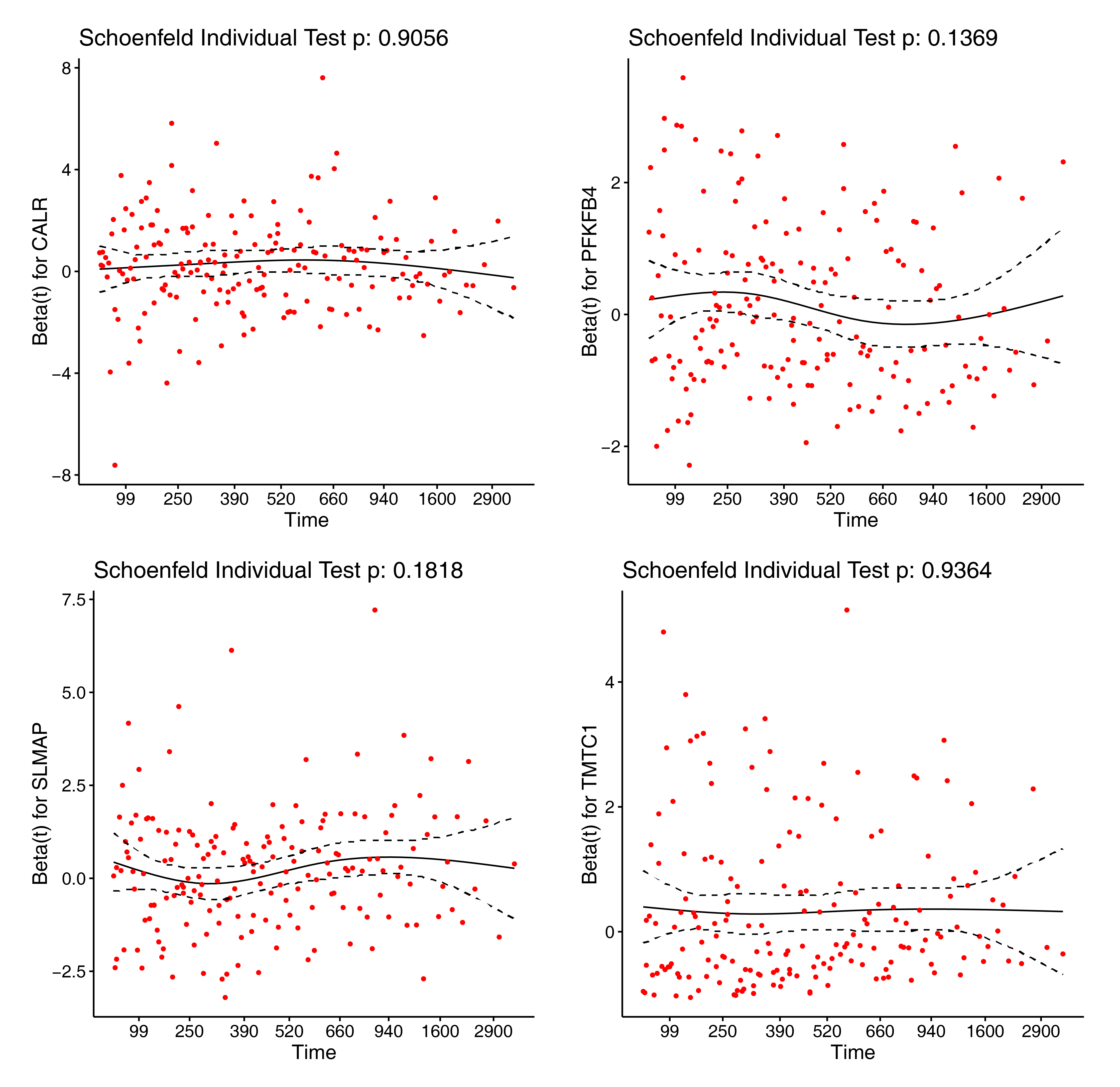

Supplement: Supplementary Figure 1 — Analysis flowchart. [file DataSheet1.zip › Supplementary Information/Figure S2.tif]
